# Psychedelic neuroplasticity of cortical neurons lacking 5-HT2A receptors

**DOI:** 10.1038/s41380-025-03257-w

**Published:** 2025-09-16

**Authors:** Tyler G. Ekins, Chloe Rybicki-Kler, Tao Deng, Isla A. W. Brooks, Izabela Jedrasiak-Cape, Ethan Donoho, Omar J. Ahmed

**Affiliations:** 1 Dept. of Psychology, University of Michigan, Ann Arbor, MI 48109 USA; 2 Michigan Psychedelic Center, University of Michigan, Ann Arbor, MI 48109 USA; 3 Neuroscience Graduate Program, University of Michigan, Ann Arbor, MI 48109 USA; 4 Dept. of Biomedical Engineering, University of Michigan, Ann Arbor, MI 48109 USA; 5 Center for Computational Medicine & Bioinformatics, University of Michigan, Ann Arbor, MI 48109 USA

**Keywords:** Neuroscience, Molecular biology

## Abstract

Classical psychedelic drugs show promise as a treatment for major depressive disorder and related psychiatric disorders. This therapeutic efficacy stems from long-lasting psychedelic-induced neuroplasticity onto prefrontal cortical neurons and is thought to require the postsynaptic expression of serotonin 2A receptors (5-HT_2A_R). However, other cortical regions such as the granular retrosplenial cortex (RSG) – important for memory, spatial orientation, fear extinction, and imagining oneself in the future, but impaired in Alzheimer’s disease – lack 5-HT_2A_R and are thus considered unlikely to benefit from psychedelic therapy. Here, we show that RSG pyramidal cells lacking postsynaptic 5-HT_2A_ receptors still undergo long-lasting psychedelic-induced synaptic enhancement. A newly engineered CRISPR-Cas-based conditional knockout mouse line reveals that this form of psychedelic-induced retrosplenial plasticity requires presynaptic 5-HT_2A_ receptors expressed on anterior thalamic axonal inputs to RSG. These results highlight a broader psychedelic therapeutic utility than currently appreciated, suggesting potential for augmenting RSG circuit function in Alzheimer’s disease, post-traumatic stress disorder, and other neuropsychiatric conditions, despite the lack of postsynaptic 5-HT_2A_ receptors.

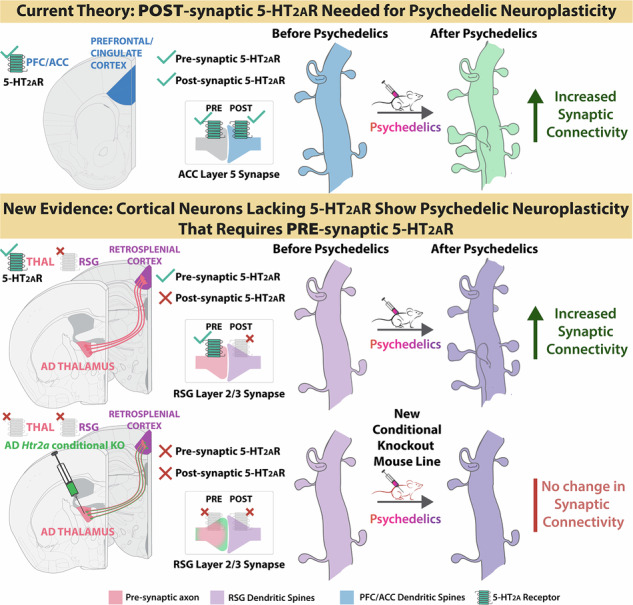

## Introduction

Classical serotonergic psychedelic drugs induce powerful perceptual experiences and show increasing promise for the treatment of a range of neuropsychiatric disorders [1–13]. Classical psychedelic drugs share a common affinity for serotonin 2A (5-HT_2A_) receptors [14–22]. Signaling cascades induced by activation of 5-HT_2A_ receptors result in a lasting enhancement of synaptic connections in the prefrontal cortex (PFC), and this synaptogenic effect is thought to provide a main neurobiological basis underlying psychedelic medicine [23–30]. Direct expression of 5-HT_2A_ receptors on the postsynaptic neuron, encoded by the gene *Htr2a*, is thus considered necessary for psychedelic-induced dendritic spine formation in the prefrontal cortex [11–13, 21–30].

Many pyramidal cells in cortex do not, however, express 5-HT_2A_ receptors [31–38]. As postsynaptic activation of 5-HT_2A_ receptors is considered essential for psychedelic-induced neuroplasticity, the prevailing assumption is that psychedelics would not offer therapeutic benefit in cells and regions that directly lack 5-HT_2A_ receptors [21–30]. Psychedelic medicine therefore has primarily focused on treating disorders traditionally associated with prefrontal and cingulate cortex where 5-HT_2A_ receptors are strongly expressed, including major depressive disorder and PTSD [11–13, 39–41].

The granular retrosplenial cortex (RSG) is perhaps the starkest example of a cortical region lacking pyramidal cell 5-HT_2A_ receptors. The RSG is part of the default mode network, integrating synaptic inputs from thalamic, cortical, hippocampal, and claustral sources to support functions ranging from memory, spatial orientation, and imagining oneself in the future [42–55]. In humans, damage to the retrosplenial cortex produces memory impairments and spatial disorientation, resulting in a dramatic inability to even find one’s way home [56–60]. The RSG is also one of the first regions to show hypoactivity in the earliest stages of Alzheimer’s disease, with almost all people living with advanced Alzheimer’s disease going on to experience severe spatial disorientation symptoms associated with RSG dysfunction [57, 58, 61]. The RSG also plays a critical role in the appropriate processing of fear, as well as the successful extinction of fearful memories [46, 55]. It is not known if classical psychedelics can boost synaptic activity and induce spinogenesis in 5-HT_2A_ receptor-lacking RSG principal neurons. The answer to this question is of critical importance, as it would open the possibility of using psychedelics to correct RSG-specific synaptic deficits in Alzheimer’s disease & fear-related disorders, as well as motivate attempts to treat other disorders involving neurons not directly expressing 5-HT_2A_ receptors.

Here, using genetic engineering of a new 5-HT_2A_ receptor (*Htr2a*) conditional knockout (cKO) mouse line, ex vivo intracellular physiology, pharmacology, optogenetics, cellular morphology, and single-nucleus/spatial transcriptomics, we test the hypothesis that psychedelics can increase synaptic connections of RSG neurons lacking 5-HT_2A_ receptors. The *Htr2a* cKO line allows for the synapse-specific dissection of rules governing psychedelic-induced strengthening of synaptic connectivity and reveals a presynaptic, but not postsynaptic, requirement for 5-HT_2A_ receptors to boost retrosplenial synaptic activity and dendritic spines. This work expands the catalog of neurological and psychiatric disorders that psychedelic medicine may be able to potentially treat via lasting synaptic enhancement.

## Methods

### Animals

All procedures and use of animals were approved by the University of Michigan Institutional Animal Care and Use Committee. Mouse lines were obtained from Jackson Laboratories unless specified. The following mouse lines were used for whole cell recordings: Ai14, Ai32, C57BL/6J, Cux2-Cre, Grp-Cre, *Htr2a*-floxed (*Htr2a*^*fl*^), Kj319. Nex-Cre, Pvalb-Ai14, Pvalb-Cre, Scnn1a-Cre, Syt6-Cre. All mice used in the experiments were bred on a C57BL6 background (Charles River). A combined total of 321 neurons from 84 mice of both sexes between the ages of postnatal days 30–306 were used.

### Creation and validation of *Htr2a* conditional knockout mice

With the University of Michigan Transgenic Animal Model Core, we utilized the CRISPR-Cas9 system in C57BL/6 mouse embryos to introduce two LoxP sites flanking the *Htr2a* Exon 1 protein-coding region (*Htr2a*^*fl*^). Single-stranded RNA guides (Supplementary Table 1) were complexed with Integrated Data Technologies HiFi Cas9 protein (IDT # 1081060) before co-injection with donor DNA into embryos. HiFi Cas9 protein is designed for high specificity of on-target activity [62]. To validate that the LoxP insertions were successful, we performed PCR genotyping using the primers specified in Supplementary Table 1. G0 founders were bred with wild-type mates to create heterozygous G1 mice, which we bred in-house to homozygosity (*Htr2a*^fl/fl^) before use in experiments.

### RNAScope

RNAScope was used to confirm presence of *Htr2a* mRNA in prefrontal cortex (PFC) and anterior dorsal (AD) thalamus. RNAScope was also used to validate loss of *Htr2a* mRNA after viral transfection of Cre. We used three *Htr2a*^*fl/fl*^ mice injected with AAV2-Cre-eGFP into PFC (P93-162) as described in the Surgical Procedures. The virus was allowed to incubate for at least 4 weeks before the start of the RNAScope assay. Two of the three animals were deeply anesthetized with isoflurane, perfused, and the brain dissected into 10% neutral buffered formalin (Sigma #HT501128) for overnight fixation. Next day the brain was moved to 70% ethanol and used for paraffin embedding and subsequent RNAScope. One animal was deeply anesthetized with Isoflurane, decapitated, and the brain was dissected and immediately snap-frozen on dry ice and kept at −80 °C until the start of the experiment (7 days). Then, the brain was sliced on a cryostat (Leica 3050S) at 14 μm and mounted onto charged slides (Fisher Scientific #12-550-15). Mounted slices were kept overnight at −80 °C degrees and used next day for RNAScope.

Immediately prior to starting the assay, frozen slides were placed in 10% neutral buffered formalin for 1 h fixation at 4 °C, then dehydrated in series ethanol steps (50, 70, and 100%). Afterwards, a barrier was drawn around each slice using a hydrophobic pen (ACD # 310018), and all the subsequent reagents used were part of the RNAScope® Multiplex Fluorescent Reagent Kit v2 (ACD #323100) unless indicated otherwise. Endogenous peroxidase activity was then blocked with hydrogen peroxide solution, and the tissue permeabilized with protease IV.

After pretreatment, a custom *Htr2a* probe was prepared (ACD). The custom *Htr2a* probe was designed to target the floxed sequence of the *Htr2a* gene in the *Htr2a*^*fl/fl*^ mouse line. The probe was applied to slices, allowed to hybridize for 2 h at 40 °C and was then washed off using the wash buffer. The probe were then amplified using a series of three amplification steps, and fluorescent signal was developed using TSA Vivid 650 (ACD #323273). The signal development round was carried out at 40° C and consisted of the channel-specific HRP incubation, fluorescent dye application, and HRP blocking, with wash buffer washes after each step. Finally, the slices were mounted with FluoromountG with DAPI (Southern Biotech #0100-20), cover slipped, and set overnight at 4 °C. Confocal imaging was then carried out.

### Pharmacology

25CN-NBOH was purchased from Tocris Bioscience and was administered to mice via intraperitoneal (IP) injection (2, 10, or 20 mg/kg, specified throughout text), dissolved in sterile saline, brought up to a volume equaling no more than 1% of the mouse’s total bodyweight. Brief sonication was used to aid solubility. Mice were moved to single housing before injection and recorded 24–72 h post-injection. For acute pharmacology recordings, 25CN-NBOH (final concentration = 10 µM) was diluted into artificial cerebral spinal fluid (ACSF) from a stock concentration dissolved in sterile water.

### Surgical procedures

Surgical anesthesia was induced via vaporized isoflurane inhalation at 5% and then maintained at 1–3% isoflurane. Upon induction, atropine was injected subcutaneously at 0.05 mg/kg. A Physitemp (Clifton, New Jersey, USA) controller monitored and maintained body temperature at 37 °C. Ophthalmic ointment was placed on the eyes. The incision site was prepared using a wash of Nolvasan (1:40) followed by isopropyl alcohol and then a subcutaneous injection of lidocaine (1%). The skull was cleaned using Hydrogen Peroxide, then leveled using bregma and lambda. Using a digital stereotaxic coordinate system, the following injection target sites were identified: right anterodorsal (AD) thalamic nucleus for ChR2 experiments (AP = −0.6 mm, ML = ± 0.78 mm, DV = −2.5, –3.25 mm), and both AD thalamic nuclei for Cre-GFP injection. Craniectomies were performed at the target sites, and then dura at the target coordinates was removed. Micropipettes were lowered under stereotaxic guidance into the target injection site containing the ChR2 viral construct (AAV2-EF1a-DIO-hChR2(h134R)-eYFP or Cre/GFP viral construct (AAV2-hSyn-GFP-Cre), obtained from UNC Gene Therapy Vector Core. Injections of 2.0 µL total virus volume were administered via a picospritzer (0.05–0.07 µL/min). After injection, there was a 10-min period before removing the micropipette from the brain. Enrofloxacin was administered (8.0 mg/kg) after injections. Craniectomies were sealed with bone wax, the skin incision was closed with VetBond, and with antibiotic ointment placed under skin edges. Isoflurane was tapered down prior to removal, after which carprofen was administered (5 mg/kg). Mice were kept warm through an artificial heat source during the recovery period. Mice then recovered for 3–6 weeks post-injection before being used in experiments.

### Slice preparation

Brains were dissected in ice-cold sucrose-substituted ACSF, saturated with 95% O2 and 5% CO2 and containing the following (in mM): 3 KCl, 1.25 NaH2PO4, 26 NaHCO3, 10 dextrose, 234 sucrose, 0.2 CaCl2, 4 MgSO4. 300 μm coronal slices were cut using a Leica Microsystems 1200VT vibratome and placed in a high-magnesium ACSF solution at 32 °C for 30 min and subsequently rested at room temperature for at least 30 more minutes before being recorded. During experiments, slices were submerged in a recording chamber with physiological temperature (32 °C) ACSF (126 mM NaCl, 1.25 mM NaH2PO4, 26 mM NaHCO3, 3 mM KCl, 10 mM dextrose, 1.20 mM CaCl2, and 1 mM MgSO4) perfused at 3 mL/min. Slices containing retrosplenial cortex (RSC) were obtained from anteroposterior (AP) distance to bregma −1.0 to −3.7 mm; slices containing anterior cingulate cortex (ACC) were obtained from AP 2.1 to −0.5 mm; slices containing anterodorsal thalamus (AD) were obtained from AP −0.4 to −0.9 mm.

### Whole-cell electrophysiology and quality control

Neurons were visualized on an Olympus BX51WI microscope, Olympus 60× water immersion lens, and Andor Neo sCMOS camera (Oxford Instruments, Abingdon, Oxfordshire, UK). Patch electrodes were pulled from borosilicate glass (Sutter Instruments; diameter 1.5 × 0.86 mm) to a tip resistance between 4–6 MΩ. The internal solution was potassium gluconate-based and contained (in mM): 130 K-gluconate, 0.6 EGTA, 10 HEPES, 2 MgATP, 0.3 Na2GTP, 6 KCl, NaCl and 0.5% biocytin (calculated E_Cl_ = −68 mV; pH = 7.25; osmolarity = 290 mOsm). Pipette capacitance compensation and bridge balance were applied; recordings were not corrected post-hoc for liquid junction potential. In ACC, only L5 neurons with regular spiking firing patterns that did not fire rebound spikes after hyperpolarizing current injection were used in this study. In RSG, LR cells in L2/3 were identified by characteristic firing patterns as previously described [48, 49, 53]. Cells were excluded if baseline resting membrane potential was more depolarized than −55 mV (or −45 mV for AD thalamus recordings), if input resistance decreased >25% before acute pharmacology experiments, if uncompensated series resistance (Rs) was >35 MΩ, or if Δ Rs >25% during acute pharmacology experiments. All whole-cell recordings were conducted with MultiClamp 700B amplifier and digitized at 20 kHz with Digidata 1550B (Molecular Devices) for collection on a computer equipped with pClamp 10.7 software (Molecular Devices). Patch electrodes additionally contained biocytin (0.5%), which enabled post-hoc imaging (see methods below) to determine somatic position and confirm cellular morphology. In ACC, we considered L5 neurons to have cell bodies 125 to 400 microns from the L1/2 border. In RSG, L2/3 was identified visually by the densely packed granular cell layer.

### Cell filling and imaging

Internal solutions for all recorded neurons contained biocytin to enable post-hoc morphological analysis [29, 48, 49, 63, 64]. Cells were recorded for at least 15 min to allow diffusion of biocytin throughout the neuron. Upon completion of recording, the pipette was carefully retracted to enable membrane resealing and the slice was immediately transferred to 4% PFA for overnight fixation. After fixation, slices were washed in phosphate buffer solution (PBS) three times (ten minutes) and incubated in PBS containing 0.4% triton, fluorescent nissl (NeuroTrace 435/455), and 1:1000 streptavidin conjugated Alexa Fluor 647 at 4 °C (48 h). Afterwards, slices were washed in PBS, mounted with FluoromountG and allowed to rest for at least 24 h before imaging. Imaging was conducted with a Zeiss Axio Image M2 microscope equipped with LSM 700 confocal system using a 63× oil objective lens as z-stacks with z-step of 0.4 µm. Neurons were then reconstructed from these files using NeuTube 1.0 software.

### Spine classification and density analysis

Spines were analyzed by an experimenter blinded to experimental conditions. For each neuron imaged, a representative main apical dendrite as well as apical tuft dendrites we imaged using a Zeiss Airyscan system. Dendrite lengths were measured in FIJI ImageJ. Spine counts were tracked using the Cell Counter plugin in FIJI. Cells that filled uniformly and had no major cut off branches were selected for further spine analysis. Z-stacks of the dendrites were obtained using a Zeiss Airyscan confocal microscope system. The z-stacks were processed in ImageJ, and the spines manually identified and counted with the “cell counter” plugin based on visual inspection. Spines are defined by visibly clear protrusions on dendrites. Up to 5 apical dendrites and up to 5 basal dendrites were selected for each neuron, and each dendrite was independently analyzed. To enable a reliable spine density measurement, branches shorter than 40 microns were not included. Note that LR neurons have fewer, thinner, and smaller dendritic branches than RS cells. The spine density for each dendrite was calculated as the number of spines on the segment divided by the length of the dendrite. For spine reconstructions, max projections of Z-stacks were obtained from the high resolution 3-D images for each group, and 5 um scale bars are added to the images with the ‘Scale Bar’ tool from ImageJ. One representative dendrite was then selected from each group and manually traced with Procreate.

### Analysis of spontaneous synaptic activity

Voltage-clamp recordings were conducted at a holding potential of −70 mV. sEPSC recordings were taken in 30-second sweeps with a brief (250 ms) hyperpolarizing test pulse (−5 mV) at the start to monitor Rs and Rin throughout the experiment. Spontaneous excitatory post-synaptic currents (sEPSCs) were analyzed by an experimenter blinded to experimental conditions using Easy Electrophysiology (version 2.4.0) in 29-second sweeps (the first second of each sweep contained the test pulse and was thus discarded). Putative events were identified with threshold-based detection (negative peak direction, 5 ms local maximum period, 30 ms decay search period, 8 pA threshold, 10 ms search period, 1-ms averaged baseline, curved baseline and threshold). Events were manually inspected, and noise events were rejected to eliminate false positive events. Frequency was determined for each sweep by diving the total number of events per sweep by 29 s, amplitude was computed by averaging all events for each sweep, half-width and decay time constant were determined from fitting an exponential to the averaged event for each sweep. During acute pharmacology experiments, sEPSCs are recorded for two consecutive sweeps in standard ACSF, immediately before switching to the drug-containing ACSF and recording sEPSCs for 20 more sweeps. In some cases, optogenetic stimulation (described below) was additionally used during acute pharmacology experiments. Stimulation occurred after the test pulse, and thus the first 2 s of every sweep were discarded for sEPSC analysis.

### Analysis of optogenetic-evoked synaptic activity

Optogenetic brain slice experiments were conducted on the same rig set-up using a 5500 K white light-emitting diode (LED; Mightex; maximum power of 14.47 mW measured at the slice focal plane). Synaptic responses to optical stimulation of the ChR2-expressing thalamic axons were measured from postsynaptic retrosplenial neurons recorded under whole-cell current-clamp or voltage-clamp conditions while optically stimulating L3. Synaptic responses were obtained from 10 Hz stimulation (10 pulses, 1 ms pulse-width, 4–5 trials). During voltage-clamp pharmacology experiments, after establishing a baseline response (1 ms pulse-width, 30 s sweeps), 25CN-NBOH was bath-applied for 10 min and RMP and evoked EPSC amplitude continued to be recorded under identical stimulation parameters. After the 10-min perfusion, the 10 Hz voltage clamp synaptic responses were again recorded (10 pulses, 1 ms pulse-width, 5 trials). The amplitude of the first pulse (pA), and the area (pA*ms) of each pulse (50 ms) were analyzed. To determine short-term synaptic transmission dynamics, each pulse in the 10 Hz train was normalized to the first pulse in both baseline and drug conditions.

### Analysis of intrinsic physiological properties

Intrinsic excitability experiments were performed and analyzed as previous described [48, 49, 53, 64]. During current clamp experiments, membrane potentials were biased to −65 mV at the start of each sweep. Firing patterns were investigated using a series of 0.6 to 1-second current injections (step size 50 pA) until 400 pA was injected or depolarization block was induced. Spikes that did not have a peak voltage reaching at least −10 mV were not counted. F-I gain was the slope of a linear regression on the number of spikes at each current injection from 0 pA to the injection at which maximum firing frequency was attained, or 400pA, whichever came first. Total spike output was the sum of all spikes until maximum firing frequency or 400 pA. We used Python (version 3.10.4) to analyze all intrinsic properties, loading all ABF recording files with pyABF and using the packages NumPy, pandas, and matplotlib. For spike properties, we defined threshold as the voltage where the slope trajectory (dV/dT) reached 10 mV/ms. Amplitude was determined by the voltage difference between the threshold and the peak. Half-width was measured at the voltage corresponding to half the spike amplitude. Maximum rise slope was the maximum of dV/dt and maximum decay slope was the minimum of dV/dt. These properties were measured for the first spike evoked by the current injection. Adaptation index was computed at approximately twice the rheobase current from the 1-second current injection and was the number of spikes in the second half of the sweep subtracted from the number of spikes in the first half, divided by the total number of spikes. Input resistance (Rin), input capacitance (Cin), and membrane time constant (TC) were assessed with a series of small hyperpolarizing current steps (typically −10 to −20 pA). Rin was computed using the voltage difference between the maximum response during the current injection and the mean voltage of the 100 ms prior to the current injection, divided by the injected current. TC was computed by fitting an exponential decay curve to the voltage trace from the start of the current injection to the maximum voltage response. Input (membrane) capacitance (Cin) was calculated using the relationship Cin = TC/Rin. Hyperpolarizing current injection can produce a noticeable “sag” in some neurons, which indicates presence of a hyperpolarization-activated cation current (I_h_) that activates after the initial hyperpolarization peak. Sag ratio was computed as the maximum response amplitude divided by the mean response amplitude of the last 50 ms of the current injection. Response amplitudes were computed relative to the baseline voltage, computed as the mean of the 100 ms prior to the current injection.

### Analysis of transcriptomic data

We obtained MERFISH data from the Allen Institute for Brain Science’s publicly available MERSCOPE v1 Whole Brain dataset [65]. This dataset’s coronal slices are spaced 200 μm apart and are numbered in ascending order from posterior to anterior. We selected slice number 36 for visualization of retrosplenial cortex, slice 50 for anterior cingulate cortex, slice 33 for AD thalamus, and slice 31 for dorsal subiculum. The thalamic glutamatergic clusters corresponding to different nuclei identified in Yao et al., 2023 were used here. To enable analysis of dorsal subiculum, we restricted to clusters 384, 385, 386, 479, 481, 482 from slices 29 to 36.

We obtained snRNA-seq data from the Allen Institute’s publicly available Mouse Whole Cortex and Hippocampus 10× dataset [66]. Using these identified clusters, we first analyzed expression of *Htr2a* in all glutamatergic neurons from each dissected cortical area. Next, we selected cells with the region labels “RSP” and “ACA”, to filter for cells from retrosplenial cortex and anterior cingulate cortex, respectively. For all violin plots and statistical comparisons, we grouped cells according to their existing cluster annotations. Cells were grouped into putative layers according to the layer annotation in their cluster label. We considered clusters with the following layer annotations as L2/3: (“L2 IT”, “L2/3 IT”); these clusters as L5: (“L4 RSP-ACA”, “L4 IT CTX”, “L4/5 IT CTX”, “L5 IT CTX”, “L5 PT CTX”, “L5 PPP”, “L5/6 IT CTX”, “L5/6 IT TPE-ENT”), and these clusters as L6 (“L6 IT CTX”, “L6 IT ENTl”, “L5/6 NP CTX”, “L6 CT CTX”, “L6b CTX”, “Car3”). We additionally used the retrosplenial-specific cluster (“133_L2 IT RSPv-POST-PRE”) as “L2/3 LR”, based on previously identified correspondence of this transcriptomic cluster to the unique low rheobase (LR) neurons found in L2/3 of granular retrosplenial cortex [48, 49, 53, 54]. UMAPs were generated with the umap-learn package in Python.

### Statistical analysis

Statistical analyses were performed in GraphPad Prism (version 10.0.1). Data were tested to determine whether they were normal, lognormal, or otherwise distributed. Spine counts and sEPSC frequency/amplitude distributions were found to be lognormally distributed, and were log transformed before statistical testing. All additional details on precise statistical tests used are provided in the figure legends.

### Scientific illustrations

Illustrations for figures were created using Adobe Illustrator. The dendritic spine drawing used in the graphical abstract was modified from SciDraw (scidraw.io).

## Results

### Psychedelics enhance synaptic connectivity in retrosplenial cortex

**Fig. 1:**
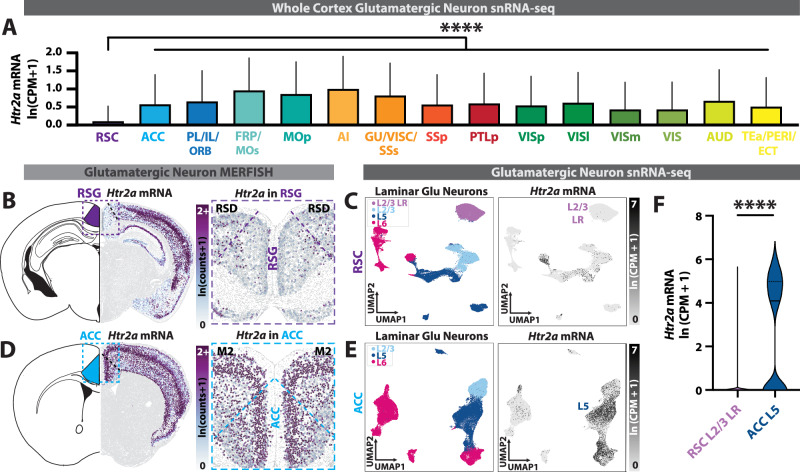
Identification of 5-HT_2A_R-lacking neocortical glutamatergic neurons. **A** *Htr2a* mRNA expression (snRNA-seq) in glutamatergic neurons across neocortical areas. Retrosplenial cortex expresses significantly less *Htr2a* mRNA than all other areas (F_(14, 810387)_ = 4019, P < 10^−15^, one-way ANOVA; for multiple comparisons with Dunnett correction between retrosplenial (RSC) and every other cortical area, P < 10^−9^). **B** *Left*, Schematic of a coronal slice containing granular retrosplenial cortex (RSG). *Right*, *Htr2a* mRNA expression in retrosplenial glutamatergic neurons from a representative MERFISH section. Note the lack of *Htr2a* expression in RSG L2/3 neurons. **C** *Left*, UMAP of retrosplenial glutamatergic neurons from snRNA-seq and color-coded by layer. *Right*, expression of *Htr2a* mRNA in retrosplenial glutamatergic neurons. **D** *Left*, schematic of a coronal slice containing anterior cingulate cortex (ACC, blue). *Right*, expression of Htr2a mRNA in anterior cingulate (ACC) glutamatergic neurons from a representative MERFISH section. **E** *Left*, UMAP of ACC glutamatergic neurons from snRNA-seq, color-coded by layer. *Right*, *Htr2a* mRNA expression in ACC glutamatergic neurons. Note the high expression in ACC L5 neurons. **F** RSG LR glutamatergic neurons express significantly less *Htr2a* mRNA compared to ACC L5 glutamatergic neurons (t_(30535)_ = 98.2, P < 10^−15^, two-tailed unpaired t-test). MERFISH and snRNA-seq data sourced from Allen Institute databases [65, 66]. Bar plots represent mean + standard deviation. ****: P < 0.0001.

We first used multiplexed error-robust fluorescence in situ hybridization (MERFISH) and single-nucleus RNA sequencing (snRNA-seq) datasets [65, 66] to investigate expression of *Htr2a* in glutamatergic neurons across neocortical areas. We found that retrosplenial cortex (RSC) has the lowest *Htr2a* expression of the entire neocortex (Fig. 1A). In particular, the defining and most ubiquitous cells of the granular RSC (RSG) – the RSG-specific L2/3 low rheobase (LR) neurons [48, 49, 53, 54] – are almost entirely void of *Htr2a* expression (Fig. 1B, C). We repeated this analysis for ACC glutamatergic neurons and found widespread expression of *Htr2a* in L5 neurons (Fig. 1D, E). Population analyses confirmed that RSG LR neurons express very little *Htr2a* mRNA and ACC L5 neurons express a dramatically and significantly higher amount (Fig. 1F), consistent with previous work indicating high 5-HT_2A_ receptor expression in frontal cortex and low expression in retrosplenial cortex [32, 34–38].

**Fig. 2:**
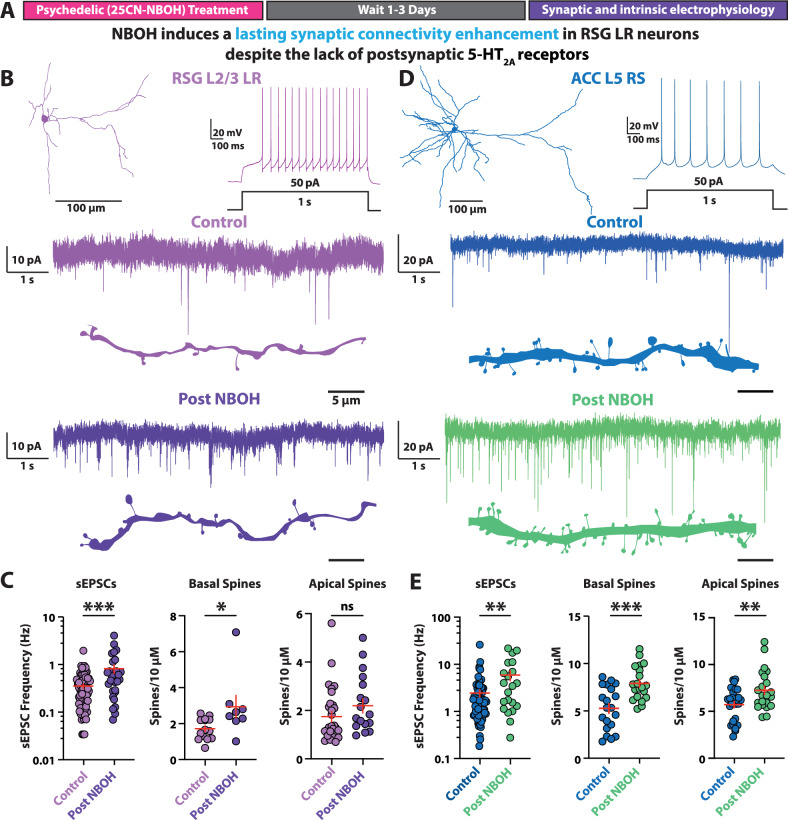
Psychedelic-induced synaptic enhancement onto neocortical neurons. **A** Experimental timeline. Mice are given a single dose of 25CN-NBOH (2 mg/kg) via intraperitoneal (IP) injection. After 1–3 days acute brain slices are taken and intrinsic and synaptic electrophysiology properties of RSG L2/3 LR pyramidal neurons and ACC L5 RS pyramidal neurons are recorded. **B** *Top*, RSG LR neuron morphological reconstruction and voltage trace showing firing pattern of RSG L2/3 LR pyramidal neuron. *Bottom*, current trace showing spontaneous excitatory postsynaptic currents (sEPSCs) and dendritic spine tracings of control (violet) and NBOH (dark purple) treated LR neurons. **C** NBOH treatment significantly increases LR sEPSC frequency (*left*; t_(99)_ = 3.552, SEM Control = 0.0357, SD Control = 0.3032, SEM Drug = 0.0872, SD Drug = 0.4930, P = 0.0006, two-tailed unpaired t test) and density of basal dendrite spines (*middle*; t_(20)_ = 2.392, SEM Control = 0.1590, SD Control = 0.5950, SEM Drug = 0.6314, SD Drug = 1.786, P = 0.0267, two-tailed unpaired t test), but not apical dendrite spines (*right*; t_(42)_ = 1.502, SEM Control = 0.2181, SD Control = 1.133, SEM Drug = 0.2933, SD Drug = 1.210, P = 0.1405, two-tailed unpaired t test). **D** *Top*, recording schematic, voltage trace showing firing pattern of ACC L5 RS pyramidal neuron. *Bottom*, Current trace showing ACC L5 RS sEPSCs and dendritic spine tracings before (blue) and after (green) NBOH treatment. (**E**) NBOH treatment significantly increases ACC L5 RS sEPSC frequency (*left*; t_(91)_ = 2.934, SEM Control = 0.4049, SD Control = 3.436, SEM Drug = 1.408. SD Drug = 6.453, P = 0.0042, two-tailed unpaired t test) and density of basal (*middle*; t_(38)_ = 3.957, SEM Control = 0.5084, SD Control = 2.274, SEM Drug = 0.4030, SD Drug = 1.802, P = 0.0003., two-tailed unpaired t test) and apical (*right*; t_(47)_ = 2.746, SEM Control = 0.3360, SD Control = 1.746, SEM Drug = 0.4556, SD Drug = 2.137, P = 0.0085, two-tailed unpaired t test) dendritic spines. Error bars represent mean +/- standard error of the mean (SEM). *p < 0.05; **p < 0.01; ***p < 0.001; ns, not significant.

Having identified that RSG L2/3 LR neurons lack expression of 5-HT_2A_ receptors, we asked if a single dose of the 5-HT_2A_ receptor-preferring psychedelic 25CN-NBOH (NBOH) [67–70] would result in a lasting enhancement of excitatory synaptic connectivity in these neurons (Fig. 2A). The current paradigm of psychedelic-induced plasticity suggests that psychedelics should not boost synaptic connectivity onto RSG LR neurons, as LR neurons do not express 5-HT_2A_ receptors [11–13, 21–30]. However, contrary to this prevailing theory, NBOH significantly increased the frequency of spontaneous excitatory postsynaptic currents (sEPSCs) in RSG LR neurons, recorded 2–3 days post-injection (Fig. 2B, C). Psychedelic-mediated enhancement of synaptic activity occurred at all doses tested (2, 10, 20 mg/kg; Supplementary Fig. 1). No changes were observed in the sEPSC amplitude or decay kinetics (Supplementary Fig. 1). As predicted from previous PFC experiments with psychedelics [23, 25, 28–30], NBOH also induced a lasting increase in the frequency of sEPSCs in ACC L5 RS neurons (Fig. 2D, E). NBOH-induced sEPSC frequency enhancement occurred with no dependence on sex in either RSG L2/3 LR neurons (Supplementary Fig. 2) or ACC L5 RS neurons (Supplementary Fig. 3). In addition, sEPSC frequencies were similar at both 2 and 3 days post-treatment (Supplementary Fig. 4). Thus, RSG LR neurons undergo psychedelic-induced synaptic boosting despite lacking 5-HT_2A_ receptors.

To determine if the NBOH-mediated glutamatergic synaptic elevation is accompanied by neuronal structural remodeling, we quantified the density of spines – the postsynaptic physical site of excitatory synapse formation – on basal and apical dendrites of RSG LR and ACC RS neurons using biocytin intracellular labeling. NBOH treatment significantly enhanced spine density in both RSG LR and ACC RS neurons (Fig. 2B–E). Interestingly, NBOH boosted only basal dendrite spine density in RSG LR neurons (Fig. 2C), potentially reflecting the potentiation of select inputs, such as those arising from the basal dendrite-targeting anterodorsal (AD) nucleus of the thalamus [49, 71, 72]. This contrasted with ACC RS neurons, which had both apical and basal spine density elevated (Fig. 2E). Taken together, psychedelic drugs can enhance synaptic connectivity of RSG neurons not expressing 5-HT_2A_ receptors in addition to its expected boost of synapses onto ACC L5 pyramidal cells.

We also examined if psychedelic treatment induces lasting changes to intrinsic excitability in addition to the synaptic changes observed. We found that the majority of RSG LR neuron passive and active intrinsic electrophysiological properties were unchanged from controls (Supplementary Fig. 5). We observed no lasting alterations of the intrinsic excitability of NBOH treated ACC L5 RS neurons (Supplementary Fig. 6), in alignment with previous PFC studies on psychedelic drugs [27, 29]. Thus, treatment with a single psychedelic dose induces a lasting enhancement of excitatory synaptic connectivity in 5-HT_2A_ receptor-lacking RSG LR neurons but leaves intrinsic excitability largely unaffected.

### Thalamic inputs to RSG express functional presynaptic 5-HT_2A_ receptors

**Fig. 3:**
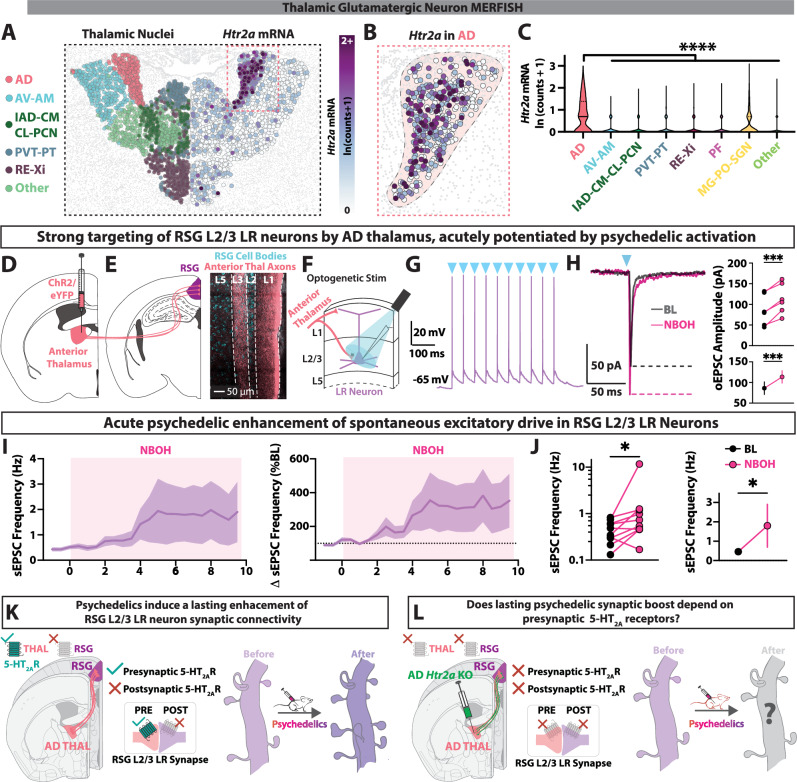
Anterodorsal thalamic cells projecting to retrosplenial cortex express 5-HT_2A_Rs. **A** MERFISH section showing thalamic glutamatergic neurons color mapped by thalamic nucleus (*left*) and *Htr2a* mRNA expression (*right*). **B** Increased magnification showing *Htr2a* expression in the anterodorsal nucleus of each hemisphere. **C** Violin plot of MERFISH glutamatergic thalamic nuclei; Anterodorsal (AD) nucleus contains significantly more *Htr2a* mRNA than all other thalamic nuclei (F_(7, 58774)_ = 896.1, P < 10^−15^, one-way ANOVA; for multiple comparisons with Dunnett correction between AD and every thalamic nucleus/group, P < 10^−15^). **D** *Right*, schematic of a brain slice containing anterior thalamic nuclei (ANT) with intracranial viral delivery of channelrhodopsin (ChR2) and enhanced yellow fluorescent protein (eYFP, pseudocolored pink here). **E** *Left*, schematic of a brain slice containing RSG. *Right*, anterior thalamic axons in RSG after intracranial viral delivery of ChR2 and eYFP into anterior thalamus. **F** Experimental recording schematic: RSG LR neurons are recorded using whole-cell electrophysiology while stimulating ChR2-expressing anterior thalamic axons. **G** Current-clamp spiking response of LR neuron upon AD axonal stimulation (L3 axon stimulation; blue triangles represent time of light pulses). **H** *Left*, Voltage-clamp response of LR neuron upon AD ChR2 stimulation before and after a 10-min perfusion with NBOH (10 μM). *Right*, quantification of baseline and in-drug AD-evoked EPSC amplitude (t_(5)_ = 8.307, SEM Baseline = 15.22, SD Baseline = 37.28, SEM Drug = 15.01, SD Drug = 36.78, P = 0.0004, two-tailed paired t test). **I** Acute NBOH increases the frequency of sEPSCs in LR neurons. sEPSC frequency across time is presented as overall frequency (Hz, left) and as percentage of baseline sEPSC frequencies (%BL, left). **J** sEPSC frequency is significantly elevated with acute NBOH application (t_(10)_ = 2.920, Baseline SEM = 0.0738, Baseline SD = 0.2333, Drug SEM = 1.109, Drug SD = 3.507, P = 0.0137, two-tailed paired t test between baseline and drug. **K** Summery of findings so far. *Left*, RSG LR neurons lack 5-HT_2A_ receptors, but AD inputs to these neurons express presynaptic 5-HT_2A_ receptors. *Right*, psychedelic treatment enhances synaptic connectivity of LR neurons. **L** Hypothesis to be tested next: does genetic elimination of presynaptic 5-HT_2A_ receptors of AD thalamus neurons abolish psychedelic-induced neuroplasticity in RSG LR neurons? snRNA-seq data are adapted from the Allen Institute [65]. Error bars represent mean +/− standard error of the mean (SEM).*p < 0.05; ***p < 0.001; ****p < 0.0001; ns, not significant.

Having found that psychedelics enhance synaptic connectivity onto 5-HT_2A_ receptor-lacking RSG L2/3 LR neurons, we hypothesized that this boost could depend on 5-HT_2A_ receptor expression on presynaptic inputs to LR neurons. Therefore, we next analyzed 5-HT_2A_ receptor expression in long-range excitatory inputs to RSG LR neurons. While granular retrosplenial cortex receives glutamatergic long-range inputs from a wide variety of cortical and subcortical sources, we have shown that LR neurons are only strongly innervated by two primary glutamatergic sources: anterior thalamus and dorsal subiculum [49] (Supplementary Fig. 7). Using MERFISH [65] and RNA sequencing (Thalamoseq) [73] datasets, we analyzed *Htr2a* mRNA in glutamatergic thalamic nuclei (Fig. 3A; Supplementary Fig. 8). We found that the anterodorsal nucleus (AD) strongly expresses *Htr2a* (Fig. 3B), with higher expression levels than all other thalamic nuclei (Fig. 3C; Supplementary Fig. 8A). We confirmed selective expression of *Htr2a* in AD thalamus using fluorescent in situ hybridization (RNAScope) experiments (Supplementary Fig. 8B). We next repeated the MERFISH analysis with dorsal subiculum glutamatergic neurons and found overall very little *Htr2a* expression (Supplementary Fig. 9A, B). Together, this combination of connectivity and transcriptomic analyses led us to the hypothesis that long-range inputs onto RSG LR cells specifically from AD thalamic cells expressing *Htr2a* are likely to be the synapses boosted by psychedelic drugs (Supplementary Fig. 9C).

To determine if AD axons synapsing onto RSG LR neurons express functional presynaptic 5-HT_2A_ receptors, we utilized pharmacology in combination with channelrhodopsin-assisted circuit mapping (Fig. 3D–F). We first recorded RSG L2/3 LR neurons while stimulating L3 anterior thalamic axons (disconnected from cell bodies), which reflect AD inputs due to the strong synaptic targeting of L3 by AD axons [46, 49, 71, 72], and found that RSG LR neurons receive strong monosynaptic input, able to drive spiking (Fig. 3G). We next recorded optogenetic-evoked excitatory post-synaptic currents (oEPSCs) from AD axons in RSG LR neurons, and subsequently bath-applied NBOH for 10 min while continuing to record AD-evoked currents (Supplementary Fig. 10A). We found that NBOH acutely increased AD→RSG LR oEPSC amplitude and modulated short-term synaptic transmission dynamics (Fig. 3H; Supplementary Fig. 10A, B). In addition, NBOH acutely increased the frequency of spontaneous EPSCs (sEPSCs) in RSG LR neurons (Fig. 3I, J).

*Htr2a*-expressing AD thalamus neurons could have 5-HT_2A_Rs in distinct cellular compartments, such as somatodendritic areas and/or axon terminals (presynaptic to RSG LR neurons) and could thus modulate different aspects of cellular neurophysiology [74]. To next determine if AD neurons express functional somatodendritic 5-HT_2A_ receptors, we directly recorded from AD neurons’ cell bodies and bath-applied NBOH (Supplementary Fig. 11A). We found that the intrinsic somatodendritic properties of AD neurons were unresponsive to psychedelic modulation of intrinsic excitability. NBOH did not significantly alter resting membrane potentials (RMP) or evoked firing rates of AD neurons (Supplementary Fig. 11B–D), in contrast to cell types in other brain regions expressing somatodendritic 5-HT_2A_ receptors [29, 75–78], but consistent with prior somatic recordings of AD neurons revealing no effect of DOI [79]. Thus, AD neurons do not express functional somatodendritic 5-HT_2A_ receptors. Additionally, NBOH had no impact on sEPSC frequency recorded in AD neurons (Supplementary Fig. 11E, F), indicating a lack of presynaptic 5-HT_2A_ receptors in the inputs to AD.

Thus, AD neurons lack functional somatodendritic 5-HT_2A_ receptors, but AD axons which target RSG LR neurons express functional 5-HT_2A_ receptors presynaptic to LR neurons, which enhance glutamatergic synaptic transmission upon acute activation. In addition, as shown in Fig. 2, psychedelics induce a lasting increase in excitatory synaptic connections in postsynaptic 5-HT_2A_ receptor-lacking RSG LR neurons. Are the presynaptic AD thalamic 5-HT_2A_ receptors required to induce this long-lasting psychedelic boost of synaptic inputs onto LR neurons in RSG (Fig. 3K, L)? This is the question we next answered by engineering a new conditional knockout mouse line.

### *Htr2a* conditional knockout reveals role of presynaptic 5-HT_2A_ receptors for psychedelic-induced synaptic enhancement

**Fig. 4:**
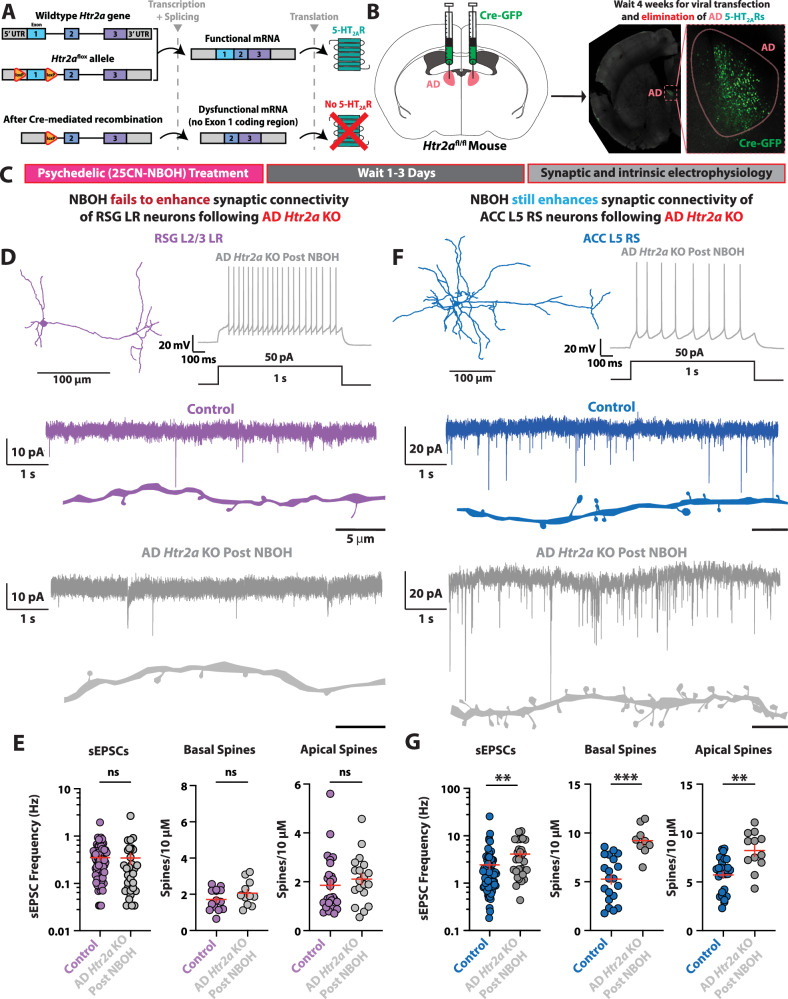
Presynaptic 5-HT_2A_Rs are required for psychedelic synaptic enhancement onto retrosplenial neurons lacking postsynaptic 5-HT_2A_Rs. **A** Strategy for manipulation of *Htr2a* gene. Schematic of *Htr2a* of wildtype (*top*), floxed allele insertion before the presence of Cre (*middle*), and after Cre-mediated recombination (*bottom*). Upon recombination, the *Htr2a* gene is unable to create viable mRNA, and 5-HT_2A_ receptor expression is lost. **B** Schematic of dual-hemisphere intracranial viral delivery of Cre and green fluorescent protein (GFP) to AD nucleus of dual-floxed *Htr2a* transgenic mice (*left*), and expression of GFP in AD neurons after transfection (*right*) **C** Experimental timeline. Mice lacking *Htr2a* expression in AD thalamus are given a single dose of 25CN-NBOH (2 mg/kg) via intraperitoneal (IP) injection. After 1–3 days, intrinsic and synaptic electrophysiology properties of RSG L2/3 LR pyramidal neurons and ACC L5 RS pyramidal neurons are recorded. **D** *Top*, LR neuron morphological reconstruction (*left*) and firing pattern after AD *Htr2a* elimination followed by psychedelic treatment (*right*). *Bottom*, sEPSCs of control (violet) and after NBOH treatment in AD *Htr2a* KO (grey) LR neurons. **E** NBOH treatment fails to increase LR sEPSC frequency (*left*; t_(102)_ = 1.559, SEM Control = 0.0357, SD Control = 0.3032, SEM Drug = 0.0872, SD Drug = 0.4930, P = 0.1221, two-tailed unpaired t test), basal dendrite spines (*middle*; t_(23)_ = 1.330, SEM Control = 0.1590, SD Control = 0.5950, SEM Drug = 0.2081, SD Drug = 0.6902, P = 0.1965, two-tailed unpaired t test), or apical dendrite spines (*right*; t_(44)_ = 0.988, SEM Control = 0.2181, SD Control = 1.133, SEM Drug = 0.2269, SD Drug = 0.9892, P = 0.3286, two-tailed unpaired t test) after AD *Htr2a* elimination. **F** *Top*, ACC L5 RS neuron morphological reconstruction (*left*) and firing pattern after AD thalamus *Htr2a* elimination followed by psychedelic treatment. *Bottom*, sEPSCs of control (violet) and after NBOH treatment in AD *Htr2a* KO (grey) LR neurons. **G** NBOH treatment still significantly increases ACC L5 RS sEPSC frequency (*left*; t_(100)_ = 3.359, SEM Control = 0.4049, SD Control = 3.436, SEM Drug = 0.6222. SD Drug = 3.408, P = 0.0011, two-tailed unpaired t test), basal spines (*middle*; t_(27)_ = 3.758, SEM Control = 0.5084, SD Control = 2.274, SEM Drug = 0.5008, SD Drug = 1.052, P = 0.0008, two-tailed unpaired t test), and apical spines (*right*; t_(37)_ = 3.359, SEM Control = 0.3360, SD Control = 1.746, SEM Drug = 0.5728, SD Drug = 1.984, P = 0.0018, two-tailed unpaired t test) after AD *Htr2a* elimination. Error bars represent mean +/− standard error of the mean (SEM).*p < 0.05; **p < 0.01; ***p < 0.001; ns, not significant.

To selectively manipulate 5-HT_2A_ receptors, we created and functionally validated an *Htr2a* conditional knockout (cKO) mouse line using Crispr-Cas9 technology (Fig. S12; Table 1). Upon introduction of the Cre protein into specific regions or cells, the dual-‘floxed’ *Htr2a* (*Htr2a*^*fl/fl*^) gene produces dysfunctional mRNA, which results in 5-HT_2A_ receptor elimination (Fig. 4A). We bilaterally delivered Cre/GFP to AD thalamic neurons of *Htr2a*^*fl/fl*^ mice to create mice that selectively lack AD 5-HT_2A_ receptors (Fig. 4B).

To determine if 5-HT_2A_ receptors located on AD axons are responsible for the acute enhancement of spontaneous excitatory drive in RSG L2/3 LR neurons, we next recorded from RSG LR cells from brain slices lacking AD 5-HT_2A_ receptors (Supplementary Fig. 13A). We found that NBOH did not significantly enhance sEPSC frequency after loss of AD 5-HT_2A_ receptors (Supplementary Fig. 13B, C). Therefore, AD axonal 5-HT_2A_ receptors are responsible for acute psychedelic enhancement of spontaneous excitatory drive in RSG L2/3 LR neurons.

We then administered a single dose of NBOH (2 mg/kg, IP), waited 1–3 days, and then conducted whole-cell electrophysiology experiments in RSG LR neurons (Fig. 4C). We found that elimination of AD thalamic 5-HT_2A_ receptors prevented the psychedelic NBOH from enhancing sEPSC frequency in RSG LR neurons (Fig. 4D, E). This effect was seen in both male and female mice (Supplementary Fig. 14). In contrast, NBOH still significantly enhanced sEPSC frequency in ACC L5 RS neurons following AD thalamic 5-HT_2A_ receptor removal (Fig. 4F, G), consistent with the lack of AD thalamic input to ACC [72] and highlighting the synapse-specificity of the conditional knockout strategy. There were no significant changes to sEPSC amplitude or kinetics of RSG LR or ACC RS neurons (Supplementary Fig. 15).

Next, we analyzed dendritic spine density in AD *Htr2a*-KO RSG L2/3 LR and ACC L5 RS neurons following NBOH treatment and compared these to control neurons. NBOH did not increase RSG LR spine density following AD *Htr2a* elimination (Fig. 4D, E). However, NBOH still significantly increased spine density in ACC RS neurons (Fig. 4F, G). Therefore, presynaptic AD thalamic 5-HT_2A_ receptors are required for psychedelic-mediated synaptic enhancement in RSG LR neurons that lack postsynaptic 5-HT_2A_ receptors. Finally, we analyzed intrinsic membrane and firing properties and found that most intrinsic electrophysiological properties were unchanged in RSG LR (Supplementary Fig. 16) and ACC RS neurons in cKO animals after NBOH treatment (Supplementary Fig. 17). Future experiments, beyond the scope of this study, utilizing conditional knockouts of serotonin receptors selectively in neurons that exclusively express somatodendritic 5-HT_2A_ receptors (but lack 5-HT_2A_ receptors in their axons/terminals) will provide additional insight into the regulation of cellular and synaptic neurophysiology by psychedelic drugs. Experiments such as those utilized here and elsewhere [23–25, 28–30, 80, 81] will help to systematically identify such neurons.

In conclusion, psychedelics can enhance synaptic connectivity onto RSG pyramidal neurons despite the lack of 5-HT_2A_ receptors on these cells. This psychedelic-induced synaptic boost in retrosplenial cortex requires presynaptic 5-HT_2A_ receptors in AD thalamic input neurons.

## Discussion

Enhancement of neuroplasticity – initiated through signaling cascades caused by postsynaptic or intracellular 5-HT_2A_ receptor activation – is thought to be a key neurobiological mechanism underlying psychedelic treatment of neuropsychiatric disorders. Based on this understanding, neurons lacking 5-HT_2A_ receptors should not undergo psychedelic-induced synapse formation [11–13, 21–30]. Here, we instead show that psychedelics can also enhance synaptic connections in cortical pyramidal neurons that lack 5-HT_2A_ receptors (Figs. 1–3), and that this form of psychedelic plasticity requires presynaptic axonal 5-HT_2A_ receptor activation (Fig. 4).

5-HT_2A_ receptors can be located postsynaptically on somatodendritic or intracellular compartments, or presynaptically on axon terminals [28, 32, 82, 83]. This location determines the precise excitability-modulating signaling cascades that are triggered by activation of 5-HT_2A_ receptors. Postsynaptic (somatodendritic) 5-HT_2A_ receptor activation induces several changes to neuronal electrophysiology, including suppression and inactivation of voltage-gated transient sodium channels [84, 85], resting membrane potential depolarization [35–38, 77] and depression of AMPA receptors [83]. In contrast, activation of axonal 5-HT_2A_ receptors induces blockade of the voltage-gated potassium channel Kv1.2, which increases excitability within axon terminals and facilitates increased excitatory glutamate release [82, 86, 87] (Fig. 3H; Supplementary Fig. 10). However, also see reference [77] for somatodendritic depolarization-mediated enhancement of sEPSC frequency. Our results show that a long-term consequence of psychedelic activation of presynaptic 5-HT_2A_ receptors is to boost synaptic activity and spine formation onto postsynaptic neurons, even when the postsynaptic neuron does not express 5-HT_2A_ receptors.

Only 26% of neurons in the mouse brain express detectable 5-HT_2A_ receptor mRNA (Supplementary Fig. 18) [65]. However, neurons typically connect to thousands of other neurons [88], indicating that many neurons lacking postsynaptic 5-HT_2A_ receptors are likely to receive at least some inputs from presynaptic neurons that do express 5-HT_2A_ receptors. Thus, the mechanistic understanding of psychedelic plasticity presented here widens the therapeutic application of psychedelic drugs. Enhancing synaptic connectivity in the retrosplenial cortex is a key example of this therapeutic potential, as RSG pyramidal neurons lack 5-HT_2A_ receptors, but are strongly innervated by 5-HT_2A_ receptor-expressing AD thalamic inputs. AD thalamus contains vestibular-dependent head direction cells, which relay this directional information to the dendrites of RSG LR neurons. These inputs are relevant to memory, vestibular-dependent spatial processing, spatial orientation, awareness, and attention [43, 49, 71, 72, 89–93]. Theoretical and experimental work shows that this synapse from AD to RSG neurons is important for supporting angular velocity encoding in the RSG, a key component of the path integration computations required to support successful spatial navigation and orientation [49, 90, 92, 94, 95]. Thus, psychedelics may have promise in treating circuit disorders involving the synapses between the anterior thalamus and retrosplenial cortex, including Alzheimer’s disease, diencephalic amnesia, wandering syndrome, and impaired extinction of fearful memories [55–61, 91–93, 96–98].

The newly created and validated *Htr2a* conditional knockout line enables the possibility of many future studies on the importance of 5-HT_2A_ receptors in mediating psychedelic-induced plasticity across the brain. This will allow the testing of novel hypotheses on the region-, cell-type-, and cellular compartment-specific functions of 5-HT_2A_ receptors, resulting in a better understanding of 5-HT_2A_ receptor physiological function and psychedelic control of neural plasticity. Such studies will help to rationally and mechanistically support the application of psychedelic drugs to treat specific disorders and will likely continue to reveal that psychedelic drugs impact far more neural circuits in the brain than previously appreciated. This promises more avenues for psychedelic treatment than currently predicted, but also suggests the need to be wary of actions on previously unexpected synapses.

## Supplementary information

Supplementary Material

## Data Availability

Data and code required to generate the figures in this manuscript are available from the corresponding author upon reasonable request. The new conditional *Htr2a* knockout mouse line is available from the corresponding author upon reasonable request.
