# Supplementary material for: Psychedelic neuroplasticity of cortical neurons lacking 5-HT2A receptors
Source: Mol Psychiatry. 2025 Sep 16;31(3):1431–42. doi: 10.1038/s41380-025-03257-w (PMC12582175; doi:10.1038/s41380-025-03257-w)
Supplement: Supplementary file 1 — Supplementary Material [file 41380_2025_3257_MOESM1_ESM.pdf]

Psychedelic neuroplasticity of cortical neurons lacking 5-HT_{2A} receptors

Tyler G. Ekins, Chloe Rybicki-Kler, Tao Deng, Isla A.W. Brooks, Izabela Jedrasiak-Cape, Ethan Donoho, & Omar J. Ahmed

SUPPLEMENTARY FIGURES

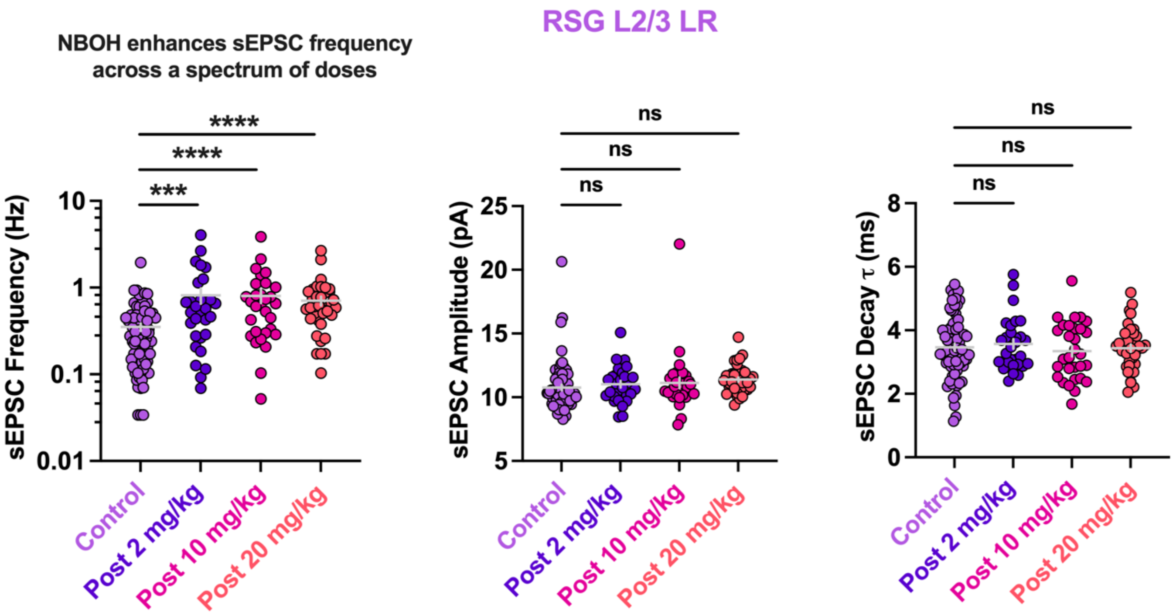

Supplementary Fig. 1. Psychedelic-induced lasting enhancement of excitatory synaptic transmission occurs across a spectrum of doses. Treatment with a single dose of 25CN-NBOH induces a lasting sEPSC frequency independently at 2, 10 and 20 mg/kg ($F_{(3, 164)}$=1.670, P=.0000008, One-way ANOVA; With Dunnett correction for multiple comparisons: Control vs 2 mg/kg, P=.0008; Control vs 10 mg/kg, P=.00009; Control vs 20 mg/kg, P=.00002). sEPSC amplitude was not altered NBOH treatment ($F_{(3, 164)}$=0.8942, P=.1621, One-way ANOVA; With Dunnett correction for multiple comparisons: Control vs 2 mg/kg, P=.7245; Control vs 10 mg/kg, P=.6845; Control vs 20 mg/kg, P=.0705). NBOH treatment also induced no lasting effects on decay time constant ($F_{(3, 164)}$=2.090, P=.7328, One-way ANOVA; With Dunnett correction for multiple comparisons: Control vs 2 mg/kg, P=.7683; Control vs 10 mg/kg, P=.9506; Control vs 20 mg/kg, P=.9878). Error bars represent mean +/- standard error of the mean. **p<0.01; ***p<0.001; ****p<0.0001; ns, not significant.

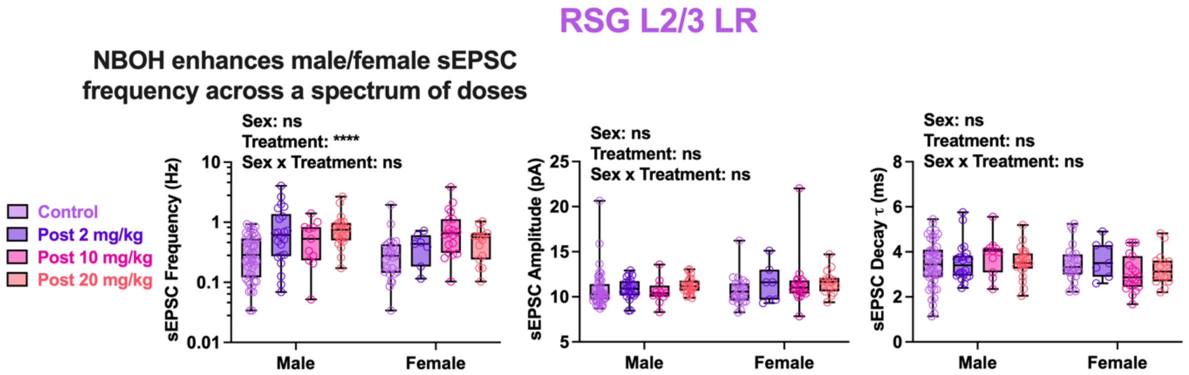

Supplementary Fig. 2. Psychedelic-induced lasting LR sEPSC frequency enhancement occurs regardless of sex. 25CN-NBOH induces a lasting sEPSC frequency in RSG L2/3 LR neurons of both sexes (Sex: $F_{(1,160)}$=0.9187, P=.3393; Treatment: $F_{(3,160)}$=8.957, P=.00002; Sex x Treatment: $F_{(3,160)}$=1.939, P=.1254; Two-way ANOVA). NBOH treatment did not impact sEPSC amplitude (Sex: $F_{(1,160)}$=1.797, P=.1820; Treatment: $F_{(3,160)}$=1.232, P=.3000; Sex x Treatment: $F_{(3,160)}$=0.6668, P=.5736; Two-way ANOVA) or decay time constant (Sex: $F_{(1,160)}$=1.194, P=.1685; Treatment: $F_{(3,160)}$=0.2363, P=.8710; Sex x Treatment: $F_{(3,160)}$=1.730, P=.1629; Two-way ANOVA). ****p<0.0001; ns, not significant.

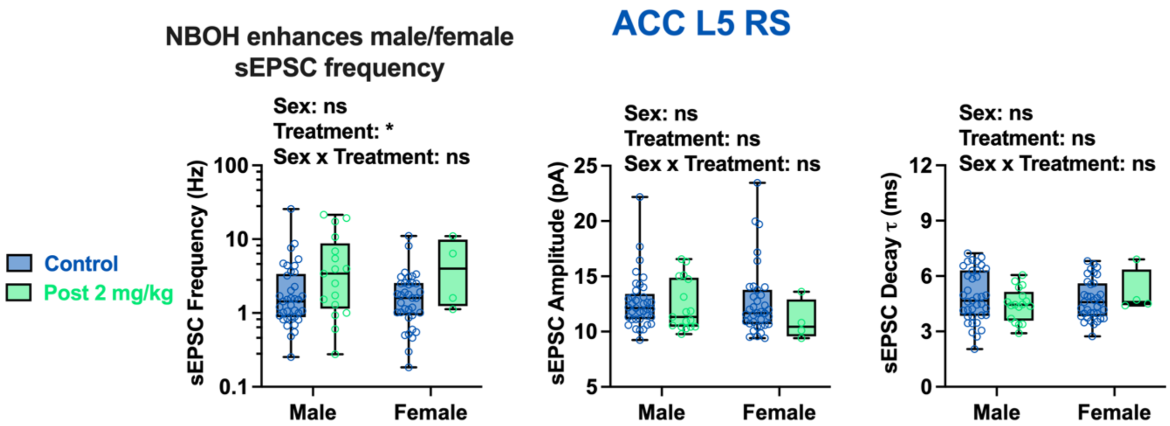

Supplementary Fig. 3. Psychedelic-induced lasting RS sEPSC frequency enhancement occurs regardless of sex. 25CN-NBOH induces a lasting sEPSC frequency in ACC L5 RS of both sexes Sex: $F_{(1,89)}$=0.0025, P=.9601; Treatment: $F_{(1,89)}$=6.161, P=.0149; Sex x Treatment: $F_{(1,89)}$=0.1123, P=.7372; Two-way ANOVA). NBOH treatment does not impact sEPSC amplitude (Sex: $F_{(1,89)}$=0.6557, P=.4202; Treatment: $F_{(1,89)}$=1.1558, P=.2152; Sex x Treatment: $F_{(1,89)}$=0.1123, P=.7372;; Two-way ANOVA) or decay time constant (Sex: $F_{(1,160)}$=0.9187, P=.3393; Treatment: Sex: $F_{(1,89)}$=0.0025, P=.9601; Treatment: $F_{(1,89)}$=6.161, P=.0149; Sex x Treatment: $F_{(1,89)}$=0.7362, P=.3932; Two-way ANOVA).*p<0.05; ns, not significant.

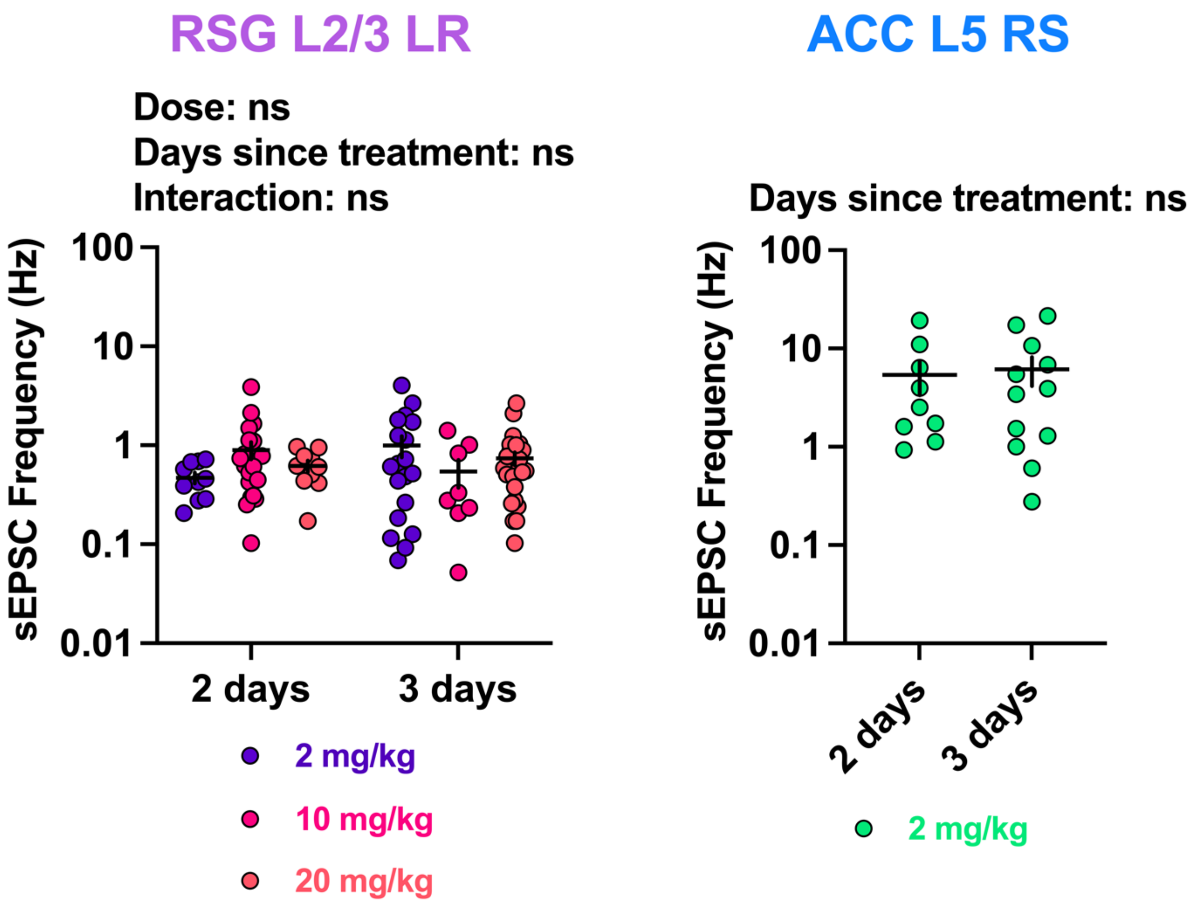

Supplementary Fig. 4. Psychedelic-induced enhancement of sEPSC frequency in RSG L2/3 LR and ACC L5 RS neurons persists through timepoints tested. Psychedelic enhancement of sEPSC frequency occurs multiple days post-dose in both RSG L2/3 LR (Dose: $F_{(2,90)}$=0.04497, P=.9560; Days since treatment: $F_{(1,90)}$=0.3572, P=.5515; Dose x Days since treatment: $F_{(2,90)}$=2.378, P=.0985; Two-way ANOVA) and ACC L5 RS Neurons ($t_{(19)}$=0.2571, P=.7998).

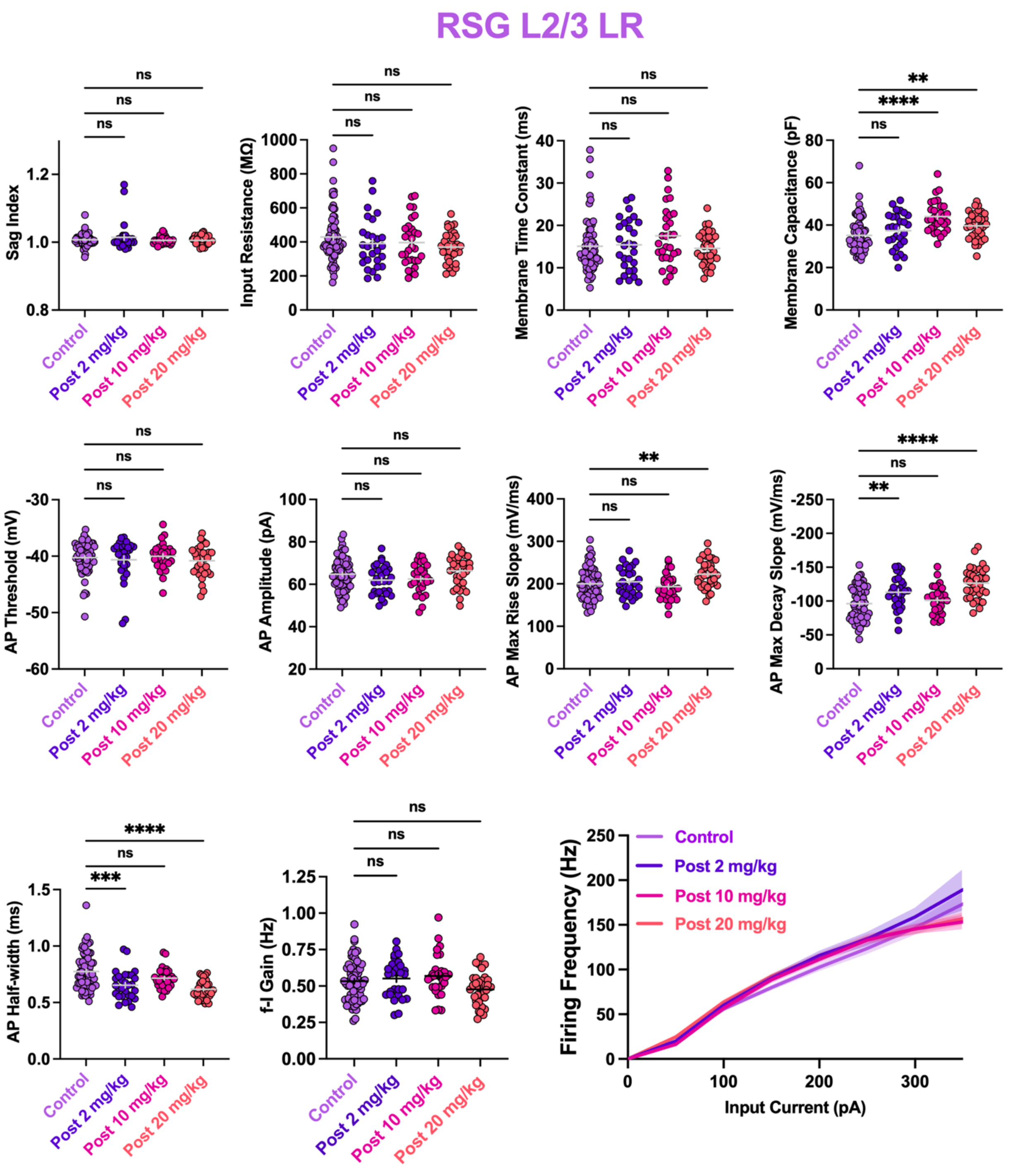

Supplementary Fig. 5. Minimal lasting psychedelic-induced changes to intrinsic excitability of RSG LR neurons. 25CN-NBOH treatment induces minimal lasting changes to intrinsic membrane and firing properties of LR neurons. A few statistically significant although small magnitude changes to certain intrinsic properties emerged mostly at higher doses. However, the vast majority of properties remain unaffected. Sag Index: $H_{(3)}$=1.867, P=.6005, control vs 2 mg/kg – P=.8053, control vs 10 mg/kg – P=.9158, control vs 20 mg/kg – P>.9999; Input Resistance: $H_{(3)}$=3.910, P=.2714, control vs 2 mg/kg – P=.5179., control vs 10 mg/kg – P=.8553., control vs 20 mg/kg – P=.2472.; Membrane time constant: $H_{(3)}$=3.849, P=.2782, control vs 2 mg/kg – P>.9999, control vs 10 mg/kg – P=.1701, control vs 20 mg/kg – P>.9999; Membrane capacitance: $H_{(3)}$=28.78, P=.000002, control vs 2 mg/kg – P=.2866, control vs 10

mg/kg – P=.000001, control vs 20 mg/kg – P=.0046; Action Potential (AP) Threshold: $F_{(3, 158)}$=0.5691, P=.6362, control vs 2 mg/kg – P=.8894, control vs 10 mg/kg – P=.9638, control vs 20 mg/kg – P=.7042; AP amplitude: $H_{(3)}$=8.220, P=.0417, control vs 2 mg/kg – P=.3268, control vs 10 mg/kg – P=.6870, control vs 20 mg/kg – P=.5033; AP Max Rise Slope: $H_{(3)}$=13.42, P=.0038, control vs 2 mg/kg – P>.9999, control vs 10 mg/kg – P>.9999, control vs 20 mg/kg – P=.0050; AP Max Decay Slope: $F_{(3,158)}$=14.76, P=.00000002, control vs 2 mg/kg – P=.0034, control vs 10 mg/kg – P=.6613, control vs 20 mg/kg – P=.000000006; AP Half-Width: $H_{(3)}$=33.72, P=.0000002, control vs 2 mg/kg – P=.0008, control vs 10 mg/kg – P=.8864, control vs 20 mg/kg – P=.0000004; Frequency-Current (f-I) Gain: $H_{(3)}$=8.616, P=.0349, control vs 2 mg/kg – P>.9999, control vs 10 mg/kg – P=.7559, control vs 20 mg/kg – P=.1501. One-way ANOVA with Dunnet correction for multiple comparisons or Kruskal-Wallis test with Dunn's correction for multiple comparisons. Error bars and shaded regions represent mean +/- standard error of the mean. *p<0.05; **p<0.01; ***p<0.001; ****p<0.0001; ns, not significant.

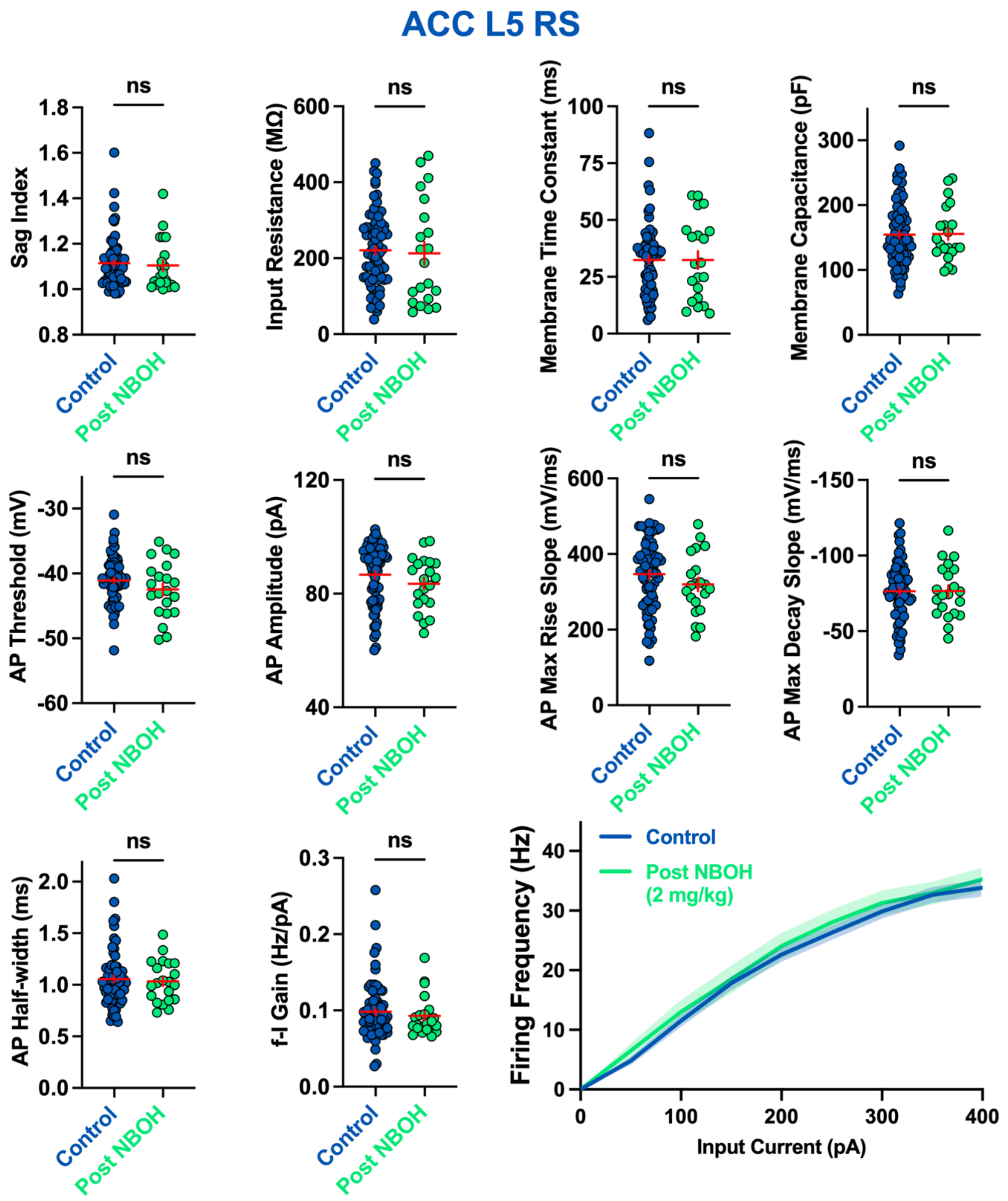

Supplementary Fig. 6. Absence of lasting psychedelic effects on intrinsic excitability of ACC L5 PCs. 25CN-NBOH treatment induces no lasting changes to intrinsic membrane and firing properties of ACC L5 RS neurons. Sag Index: $U_{(N=69,21)}$=633, P=.3867; Input Resistance: $t_{(91)}$=0.2917, P=.7712; Membrane Time Constant: $U_{(N=72,21)}$=749, P=.9527; Membrane capacitance: : $U_{(N=72,21)}$=737, P=.8660; AP Threshold: $t_{(88)}$=1.503, P=.1365; AP Amplitude: $t_{(88)}$=1.221, P=.2265; AP Max Rise Slope: $t_{(88)}$=1.221, P=.2265; AP Max Decay Slope: $t_{(88)}$=0.014, P=.9888; AP Half-Width: $U_{(N=69,21)}$=737, P=.9302; f-I Gain: $U_{(N=69,21)}$=657.5, P=.5270. Two-tailed unpaired t= test or Mann-Whitney test. Error bars and shaded regions represent mean +/- standard error of the mean. ns, not significant.

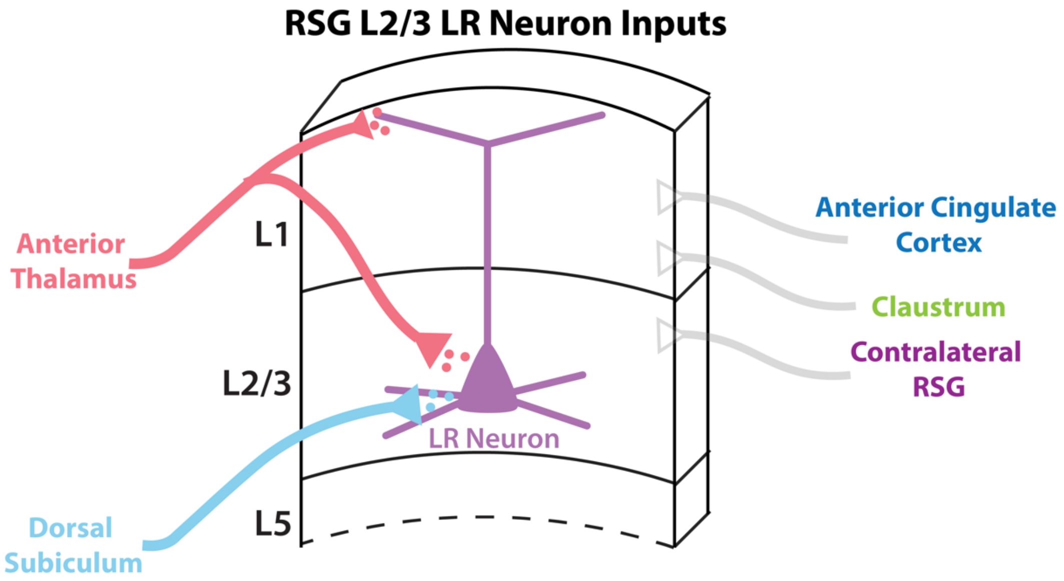

Supplementary Fig. 7. Summary of long-range excitatory inputs to RSG LR neurons. Although dorsal subiculum, anterior thalamus, claustrum, anterior cingulate cortex, and contralateral retrosplenial cortex all project to the granular retrosplenial cortex, only dorsal subiculum and anterior thalamus make strong connections with L2/3 LR neurons[44,45,48].

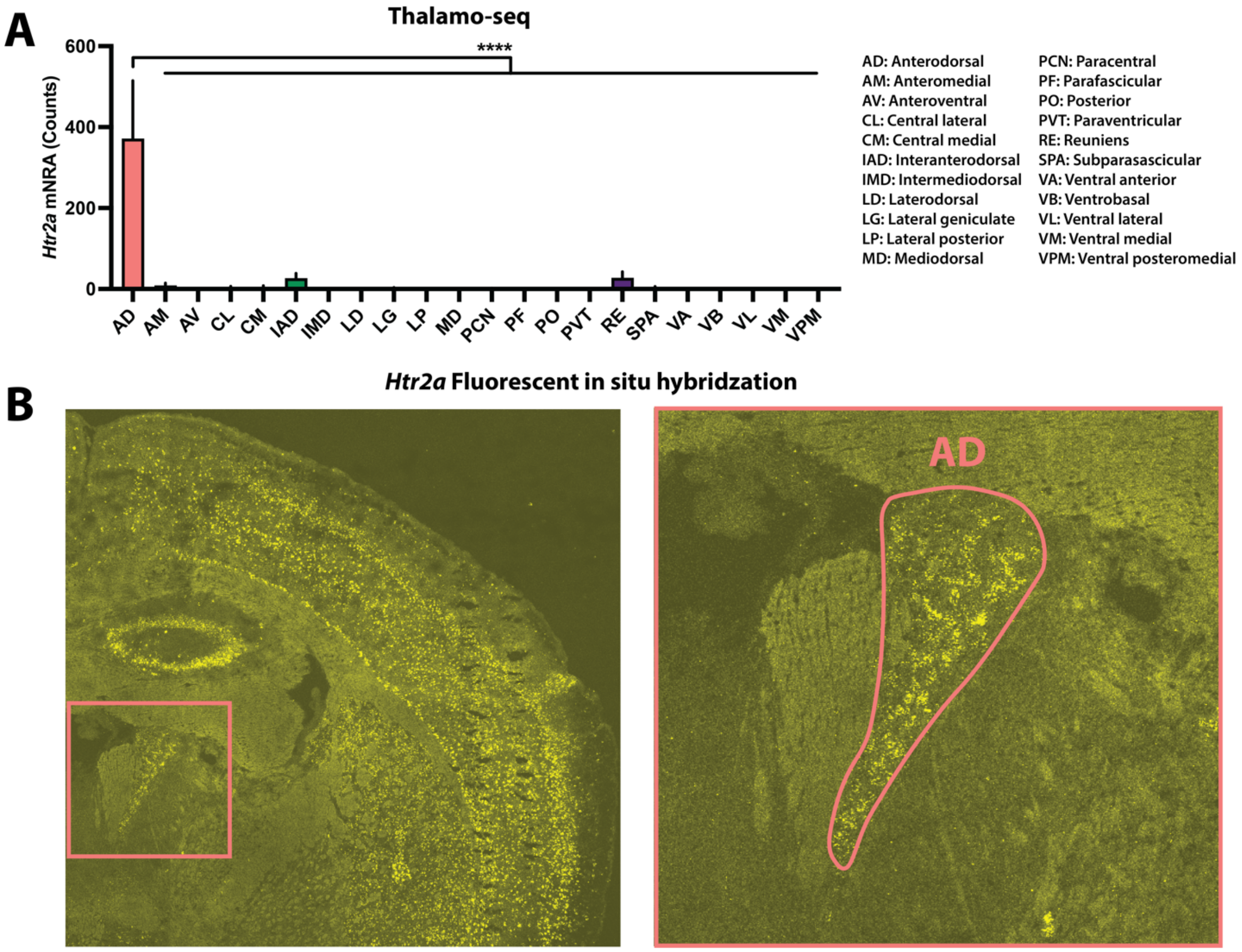

Supplementary Fig. 8. Expression of thalamic glutamatergic neuron *Htr2a* is restricted to the AD nucleus. (A) The AD nucleus contains significantly more *Htr2a* mRNA than all other thalamic nuclei ($F_{(21,98)}$ =20.52, P<10^{-14}, One-way ANOVA, For multiple comparisons with Dunnet correction between AD and every other thalamic nucleus, P< 10^{-10}). Thalamoseq data is provided by Janelia Research[73]. (B) Fluorescent in situ hybridization of Htr2a mRNA using RNAscope. AD nucleus is boxed in pink (*left*). Right side shows increased magnification in B, indicating strong Htr2a mRNA expression in AD. Error bars represent mean +/- standard deviation (SD).*p<0.05; **p<0.01; ***p<0.001; ****p<0.0001; ns, not significant.

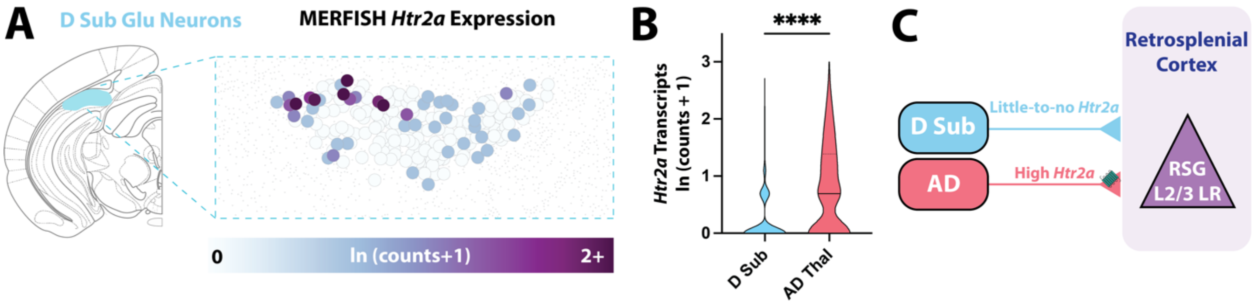

Supplementary Fig. 9. Dorsal subiculum glutamatergic neurons have low expression of 5-HT$_{2A}$ receptor mRNA. (A) *Left*, brain slice depicting dorsal subiculum (D Sub) glutamatergic neurons highlighted in blue. *Right*, *Htr2a* mRNA MERFISH expression. (B) Violin plot indicating that D Sub glutamatergic neurons express a very low amount of *Htr2a* mRNA and AD thalamus glutamatergic neurons express a significantly higher amount ($t_{(4013)}$=27.62, $P<10^{-15}$, unpaired t test). (C) Circuit diagram and *Htr2a* expression in long-range excitatory inputs to LR neurons. While LR neurons are strongly targeted by D Sub and AD Thalamus, only AD Thalamus expresses Htr2a in high amounts. snRNA-seq data from the Allen Institute[65]. ****p<0.0001.

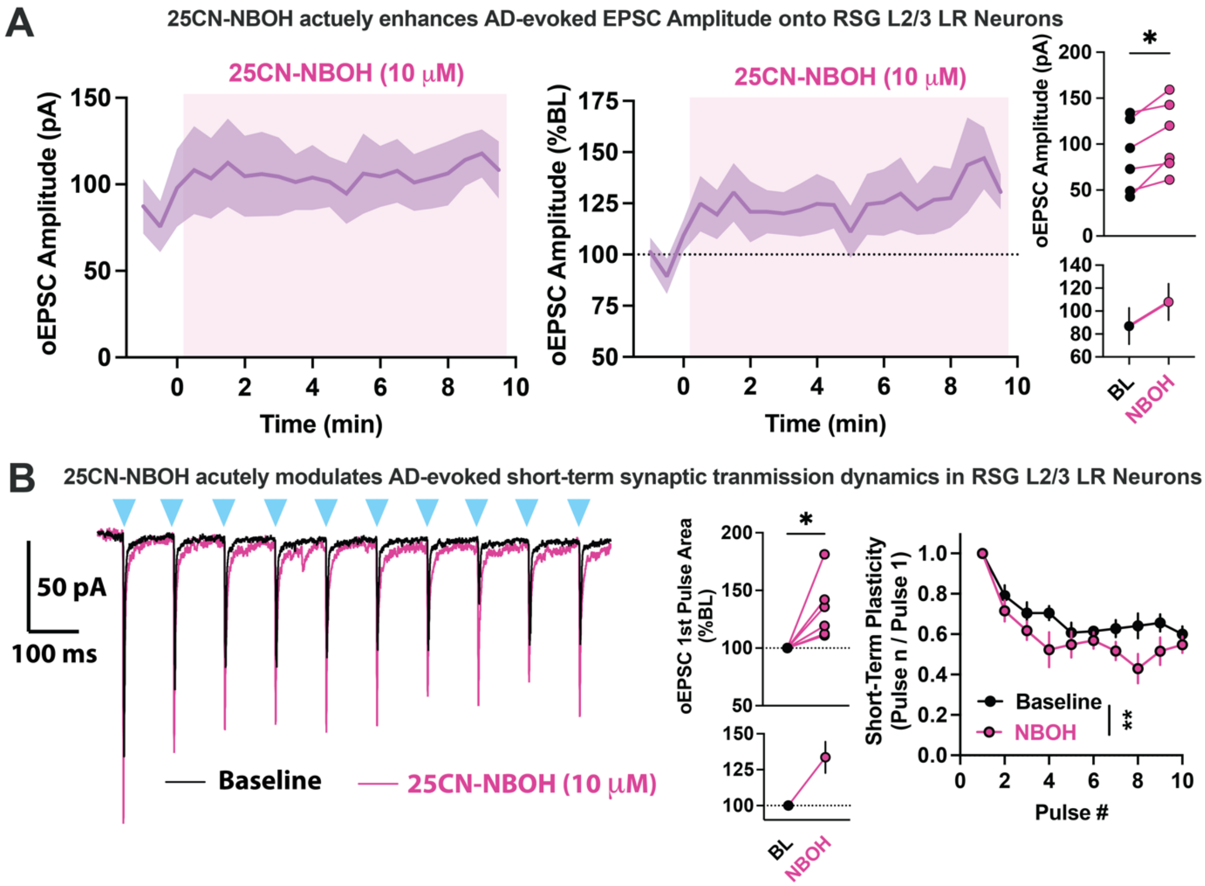

Supplementary Fig 10. NBOH acutely enhances excitatory synaptic transmission to LR neurons. (A) Bath application of NBOH acutely increases the amplitude of anterodorsal thalamus optogenetic-evoked onto LR neurons ($t_{(5)}$=3.588, P=.0157, paired t test between baseline (first 3 sweeps) and NBOH (last 8 sweeps). (B) Acute NBOH increases AD-evoked synaptic transmission, as measured by area under the curve ($t_{(5)}$=3.101, P=.0268) and increases short-term synaptic depression across a 10-Hz train ($t_{(9)}$=4.654, P=.0012, two-tailed paired t test), indicating a presynaptic site of action. Error bars and shaded regions represent mean +/- standard error of the mean.*p<0.05.

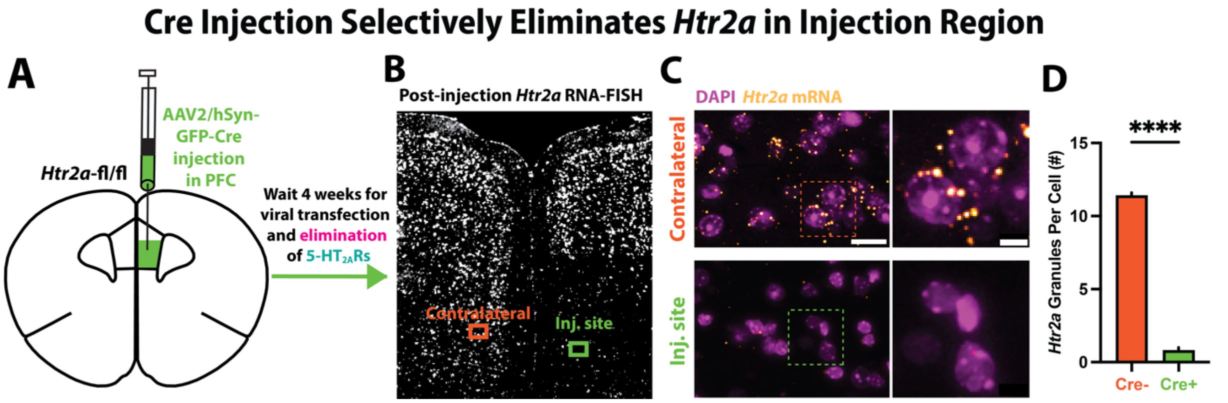

Supplementary Fig. 11. Validation of *Htr2a* conditional knockout mouse line. (A) Schematic showing injection into *Htr2a*$^{fl/fl}$ mouse brain. A virus containing Cre is injected into one hemisphere of PFC (known to contain high amounts of *Htr2a*) and followed by a 4 week wait period to allow transfection and elimination of functional *Htr2a* mRNA in the injected area. (B) *Htr2a* mRNA in PFC detected with fluorescence *in situ* hybridization (FISH). Note the lower *Htr2a* expression in the lower right injected quadrant. (C) *Left*, increased magnification of the solid orange and green boxes in B indicating lack of *Htr2a* mRNA selectively in the injection site (*bottom*). *Right*, increased magnification of the dashed orange and green boxes. (D) Quantification of *Htr2a* mRNA in L5a of control and injected PFC hemispheres indicating loss of Htr2a expression Cre injection ($U_{(N=30,\ 30)}=0$, $P<10^{-15}$, two-tailed unpaired Mann-Whitney test). Error bars represent mean +/- standard error of the mean. ****p<0.0001. Scale bars in D: 20 µm (left), 5 µm (right).

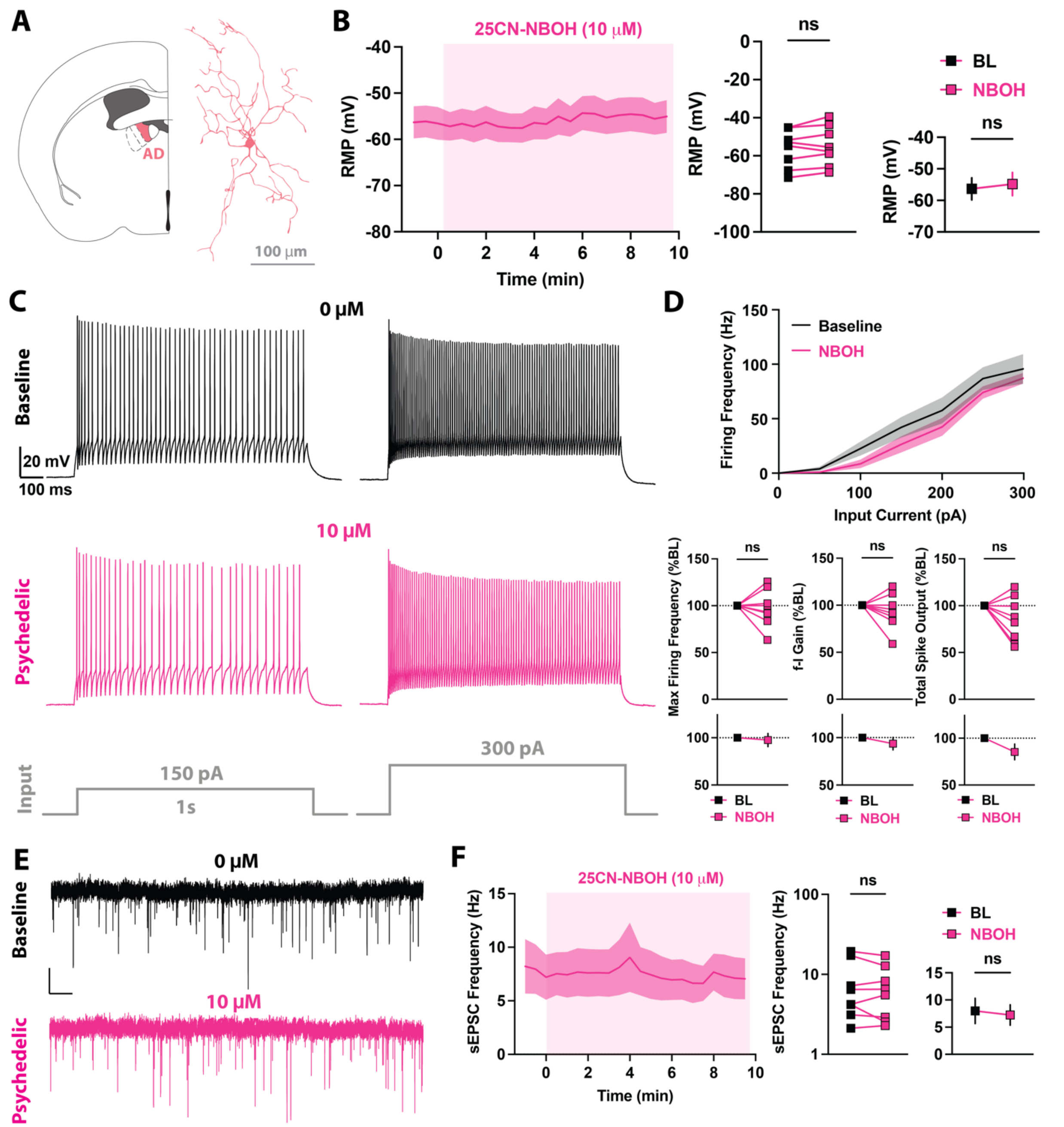

Supplementary Fig. 12. Lack of psychedelic effects on AD neuron somatodendritic electrophysiology. (A) Anterodorsal (AD) neurons were recorded and 25CN-NBOH (10 µM) was bath applied. (B) No significant changes were observed to resting membrane potential (RMP) depolarization ($t_{(7)}$=1.451, P=.1902, two-tailed paired t test). (C-D) No significant changes in evoked spiking were observed (Max Firing Frequency: $t_{(7)}$=0., P=.; f-I Gain: $t_{(7)}$=0.9544, P=.3717; Total Spike Output: $t_{(7)}$=1.742, P=.1251; two-tailed paired t tests). (E-F) No changes were observed in excitatory synaptic input onto AD neurons($t_{(7)}$=0.7138, P=.4985, two-tailed paired t test). Error bars and shaded regions represent mean +/- standard error of the mean; ns, not significant.

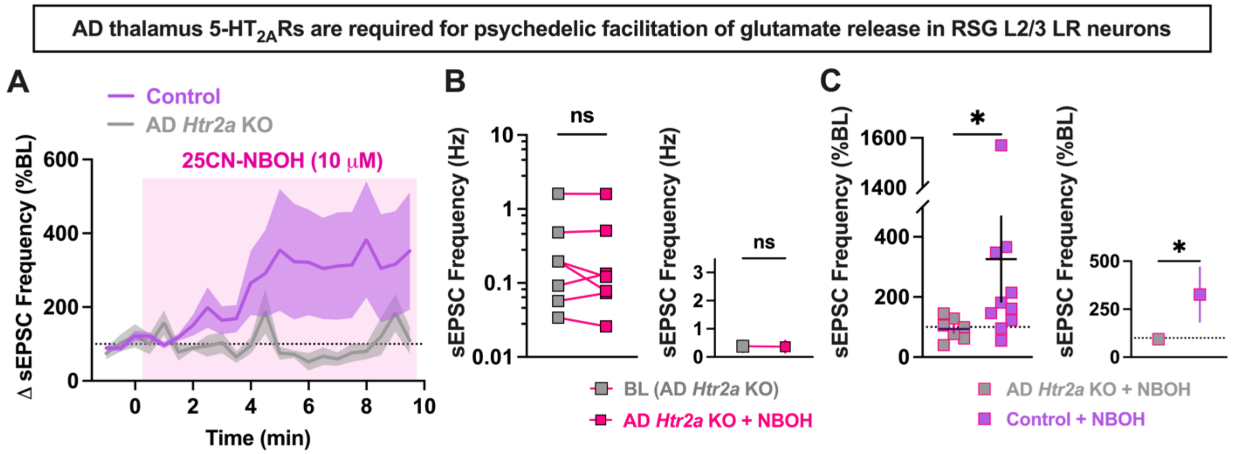

Supplementary Fig. 13 AD thalamus axonal 5-HT$_{2A}$Rs are required for psychedelic facilitation of glutamate release in RSG L2/3 LR neurons. (A) *Htr2a* mRNA was conditionally removed from AD thalamus neurons. 25CN-NBOH was acutely bath applied for 10 minutes post-baseline. (B) After knockout of AD 5-HT$_{2A}$ receptors, 25CN-NBOH (10 μM) is unable to significantly elevate sEPSC frequency in RSG L2/3 LR neurons ($t_{(6)}$=0.8347, P=.4358, two-tailed paired t test). (C) Compared to control RSG LR neurons, NBOH does not increase acute sEPSCs after loss of 5-HT$_{2A}$Rs in AD thalamus ($U_{(N=7,\ 10)}$=12, P=.0250, two-tailed Mann-Whitney Test). Error bars and shaded regions represent mean +/- standard error of the mean; *p<0.05; ns, not significant.

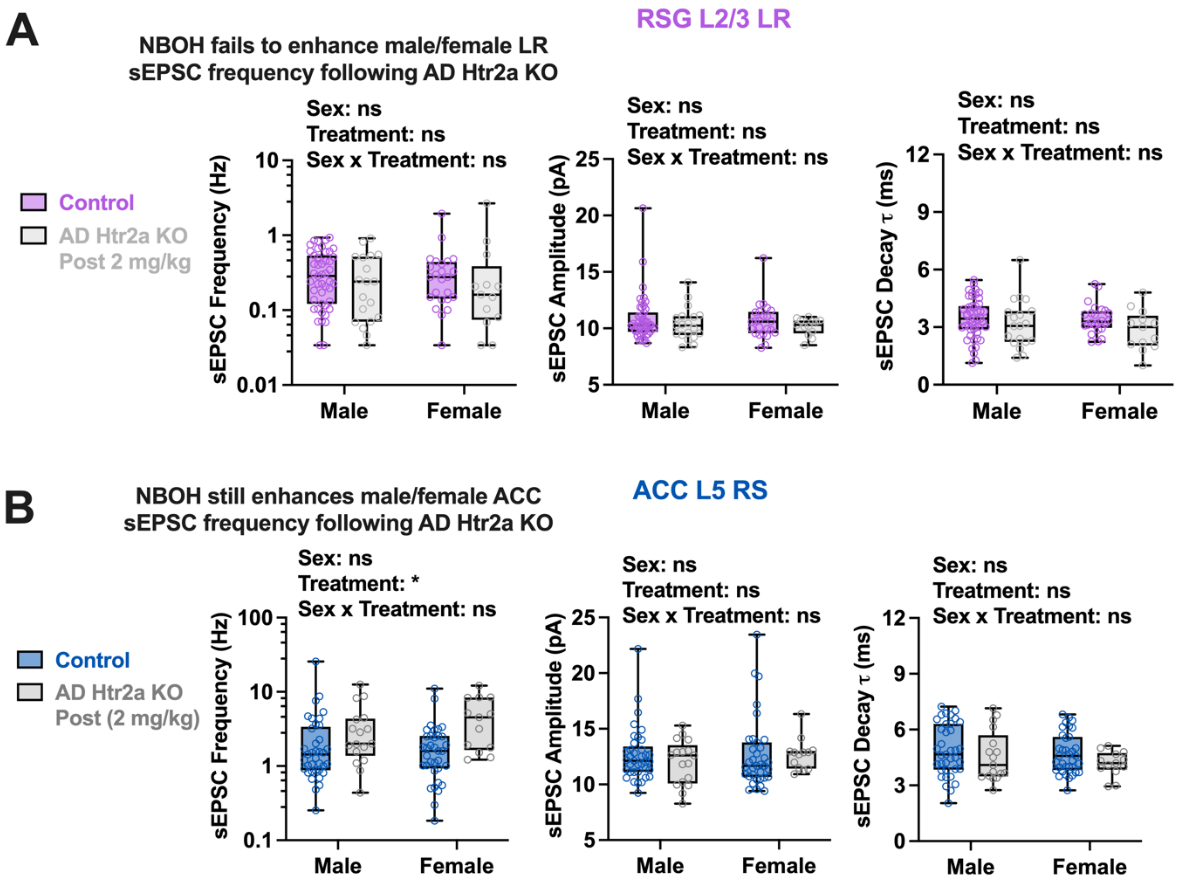

Supplementary Fig. 14. Lack of sex differences to NBOH-induced synaptic changes in AD *Htr2a* knockout. (A) NBOH treatment induces no sex-specific lasting sEPSC changes in LR neurons after AD *Htr2a* knockout (Frequency -- Sex: $F_{(1,99)}$=0.4918, P=.4848; Treatment: $F_{(1,99)}$= 0.00001, P=.9976; Sex x Treatment: $F_{(1,89)}$=0.4090, P=.5239; Amplitude -- Sex: $F_{(1,99)}$=0.2232, P=.6376; Treatment: $F_{(1,99)}$= 1.616, P=.2067; Sex x Treatment: $F_{(1,99)}$=0.0084, P=.9273; Decay Time Constant -- Sex: $F_{(1,99)}$=0.6350, P=.4274; Treatment: $F_{(1,99)}$= 3.467, P=.0656; Sex x Treatment: $F_{(1,99)}$=0.4541, P=.5020; Two-way ANOVA). (B) Following AD *Htr2a* knockout, NBOH still enhances sEPSC frequency in ACC L5 NBOH treatment but does not induce sex-specific changes and does not impact sEPSC amplitude or decay time constant (Frequency -- Sex: $F_{(1,98)}$=0.1741, P=.6774; Treatment: $F_{(1,98)}$= 5.599, P=.0199; Sex x Treatment: $F_{(1,98)}$=1.724, P=.1923; Amplitude -- Sex: $F_{(1,98)}$=0.4233, P=.5168; Treatment: $F_{(1,98)}$= 0.3575, P=.5513; Sex x Treatment: $F_{(1,98)}$=0.3369, P=.5630; Decay Time Constant -- Sex: $F_{(1,98)}$=1.200, P=.2760; Treatment: $F_{(1,98)}$= 2.655, P=.1064; Sex x Treatment: $F_{(1,98)}$=0.1382, P=.7109; Two-way ANOVA).*p<0.05; ns, not significant.

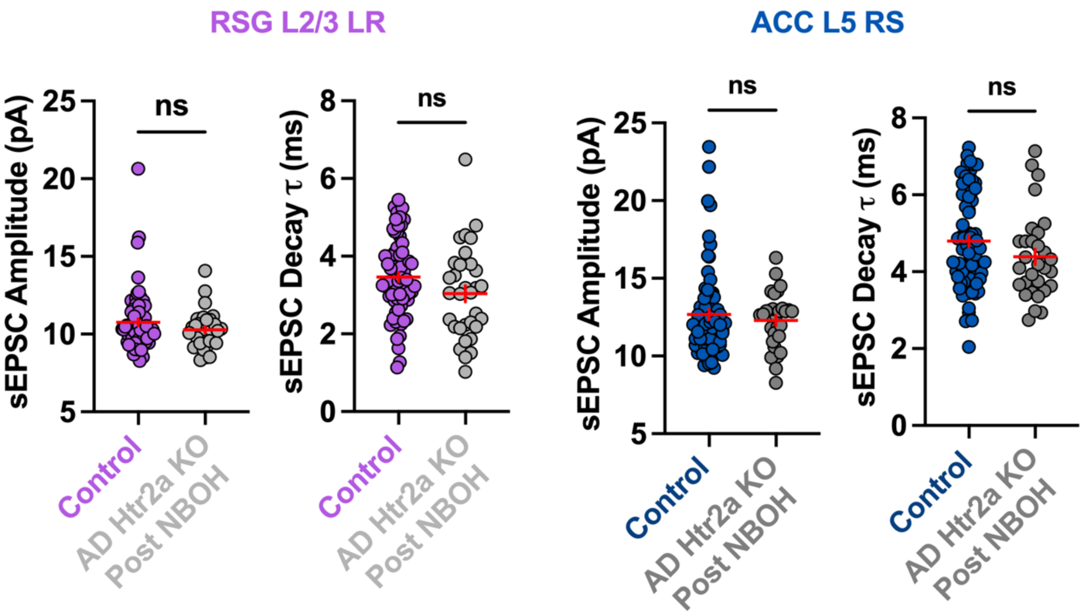

Supplementary Fig. 15. Lack of lasting NBOH-induced changes to sEPSC properties in AD *Htr2a* knockout. NBOH treatment (2 mg/kg) induces no lasting changes on sEPSC amplitude and decay time constant in LR (Amplitude: $U_{(N=72,\ 32)}$=1007, P=.3097, Two-tailed unpaired Mann-Whitney test; Decay time constant: $t_{(98)}$=0.6263, P=.5326, Two-tailed unpaired t test) and RS neurons (Amplitude: $U_{(N=72,\ 21)}$=629, P=.2461, Two-tailed unpaired Mann-Whitney test; Decay time constant: $t_{(100)}$=1.1558, P=.1224, Two-tailed unpaired t test) after AD *Htr2a* knockout. Error bars represent mean +/- standard error of the mean ns, not significant.

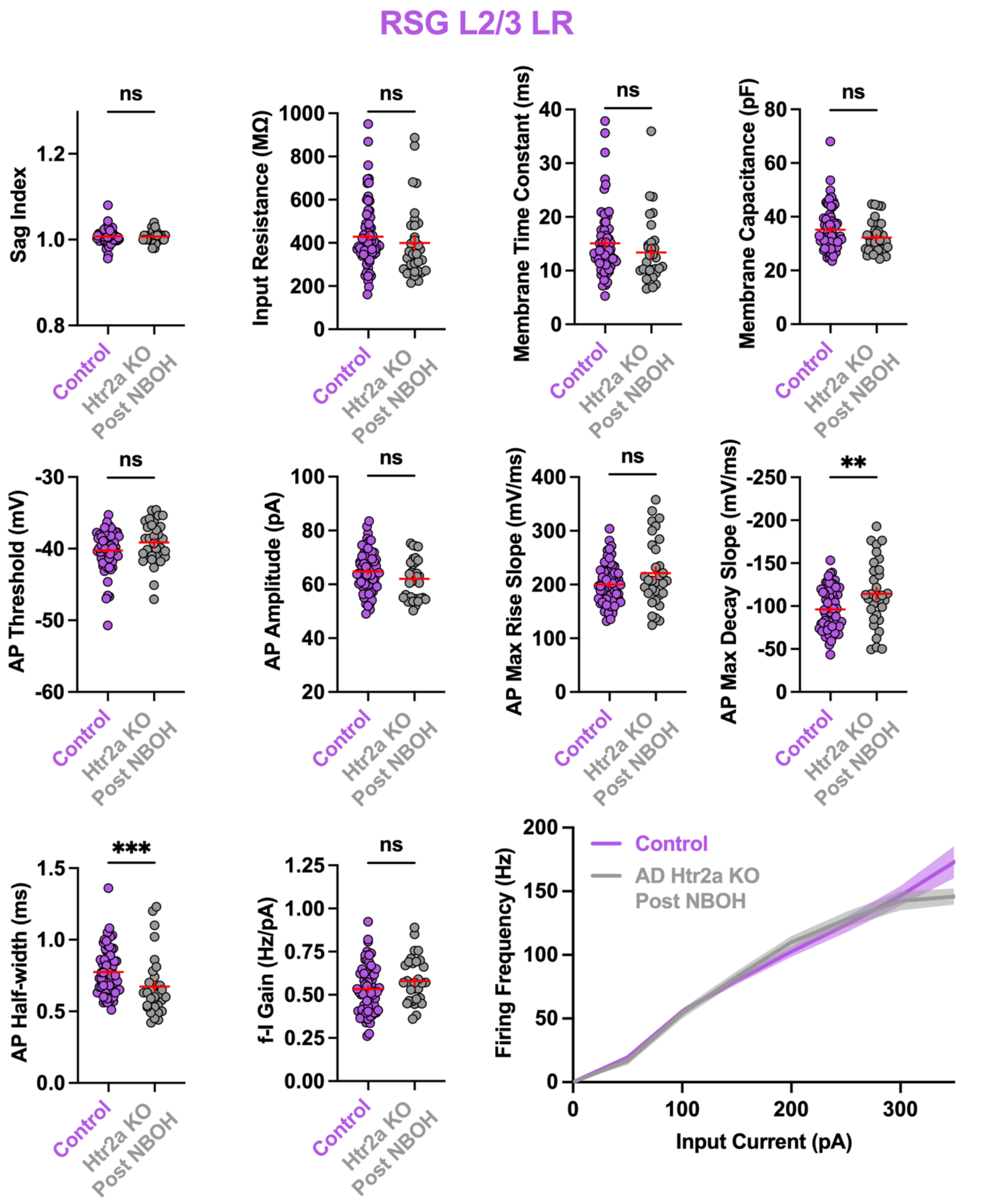

Supplementary Fig. 16 Minimal lasting NBOH-induced changes to LR neuron intrinsic excitability in AD *Htr2a* knockout. NBOH treatment induces minimal lasting changes to intrinsic membrane and firing properties of LR neurons following AD *Htr2a* knockout. It should be noted that a few statistically significant but low magnitude changes were observed. Sag Index: $U_{(N=65,31)}$=982, P=.8436**;** Input Resistance $U_{(N=70,32)}$=887.5, P=.0940**;** Membrane Time Constant: $U_{(N=70,32)}$=870.5, P=.0721; Membrane capacitance: $U_{(N=70,32)}$=858.5, P=.0593; AP Threshold: $t_{(98)}$=1.871, P=.0644**;** AP Amplitude: $U_{(N=68,32)}$=874, P=.1146; AP Max Rise Slope: $U_{(N=68,32)}$=913.5, P=.1990; AP Max Decay Slope: $t_{(98)}$=2.892, P=.0047; AP Half-Width: $U_{(N=68,32)}$=632.5, P=.0006**;** f-I Gain: $U_{(N=68,32)}$=863.5, P=.0977. Two-tailed unpaired t= test or Mann-Whitney test. Error bars and shaded regions represent mean +/- standard error of the mean. **p<0.01; ***p<0.001; ns, not significant.

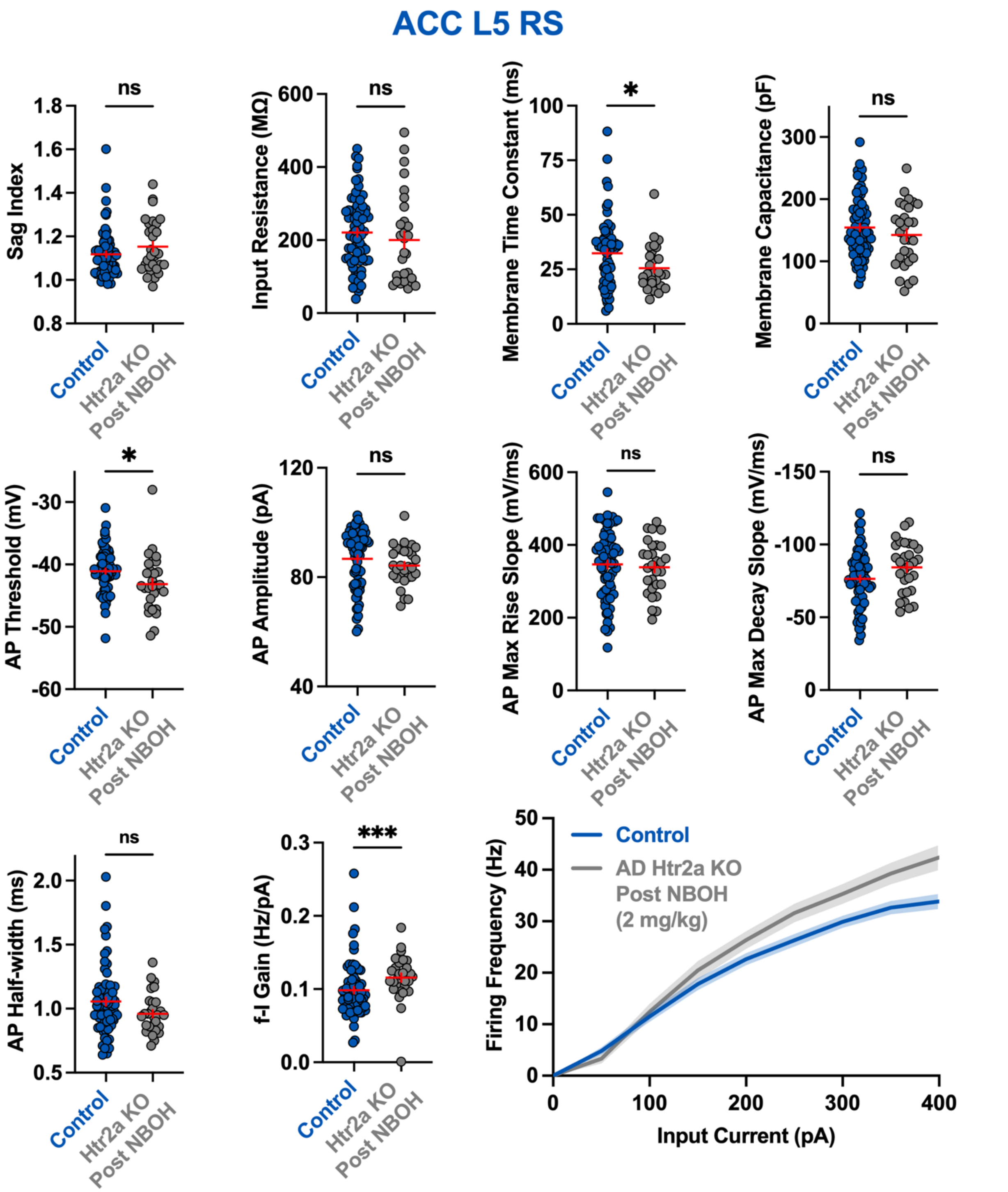

Supplementary Fig. 17. Minimal lasting NBOH-induced changes to RS neuron intrinsic excitability in AD *Htr2a* knockout. NBOH treatment induces minimal lasting changes to intrinsic membrane and firing properties of ACC L5 RS neurons following AD *Htr2a* knockout. However, some statistically significant but low magnitude changes were observed. Sag Index: $U_{(N=67,30)}$=837.5, P=.1928; Input Resistance $t_{(99)}$=0.8693, P=.3868**;** Membrane Time Constant: $U_{(N=72.28)}$=703.5, P=.0189; Membrane capacitance: $t_{(99)}$=1.083, P=.2813; AP Threshold: $t_{(96)}$=2.414, P=.0177; AP Amplitude: $t_{(96)}$=1.126, P=.2631; AP Max Rise Slope: $t_{(96)}$=0.4186, P=.6765; AP Max Decay Slope: $t_{(96)}$=1.943, P=.0549; AP Half-Width: $U_{(N=69,29)}$=774.5, P=.0788; f-I Gain: $U_{(N=69,\ 29)}$=537.5, P=.0002. Two-tailed unpaired t= test or Mann-Whitney test. Error bars and shaded regions represent mean +/- standard error of the mean. *p<0.05, ***p<0.001; ns, not significant.

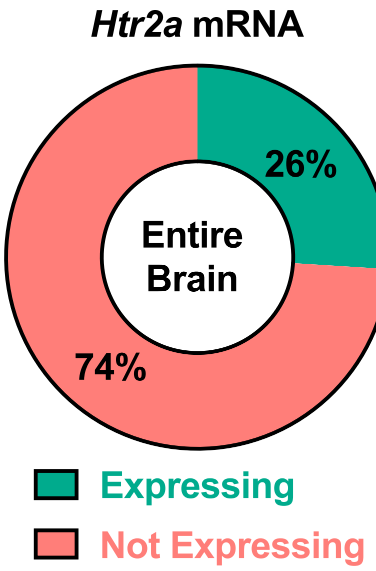

Supplementary Fig. 18 Expression of 5-HT$_{2A}$ receptor mRNA in the entire mouse brain. Only 26% of neurons across the brain express detectible *Htr2a* mRNA. Based on data from Allen Institute[65].

SUPPLEMENTARY TABLES

Genotyping PCR Primers

Target	Primer Name	Sequence (5' to 3')
3' LoxP Site	3' LoxP Forward	CCACCAACTATTTCCTGATGTCACTTG
	3' LoxP Reverse	AAAGATGTATCCATTCTAAGACACACAAC
5' LoxP Site	5' LoxP Forward	TTTTCTTCCTTAACCAGGAACACGTTTGT
	5' LoxP Reverse	TAAGCCGGAAGTTGTAGCAGATGAAG
Floxed Region	Spanning Forward	TTTTCTTCCTTAACCAGGAACACGTTTGT
	Spanning Reverse	CTCTCTCTGCAAATGTTCCAAGGTAATTG

Cas9 sgRNA and DNA Sequences

Insertion	Cas9 sgRNA (5' to 3')	Inserted DNA (5' to 3')
5' LoxP	AAUCAAGAGCCAUCACACUUCUGUAACUCUUACUA UGGAAGAGGAGAAAGCAGCCAGAGGAGCCACACA GGUCUCCGCUUCAGCAUGCCCUAGCUCCAGG	ATAACTTCGTATAATGT ATGCTATACGAAGTTAT
3' LoxP	UAGUGGAGGGACUGGGGCUCCAGGAGAGAAAUAG ACAGGAAACUUUGUUUUAGUCUUAAAAUUUUAUUU UUCUUGAGAGAAAAAAAAAAGUCAGACAGCC	ATAACTTCGTATAATGT ATGCTATACGAAGTTAT

Supplementary Table 1. *Htr2a* PCR primers and Cas9 sgRNA and DNA sequences.
